# Prediction of evolutionary constraint by genomic annotations improves functional prioritization of genomic variants in maize

**DOI:** 10.1186/s13059-022-02747-2

**Published:** 2022-09-01

**Authors:** Guillaume P. Ramstein, Edward S. Buckler

**Affiliations:** 1 Center for Quantitative Genetics and Genomics, Aarhus University, 8000 Aarhus, Denmark; 2 Institute for Genomic Diversity, Cornell University, Ithaca, NY 14853 USA; 3 USDA-ARS, Ithaca, NY 14853 USA

**Keywords:** Comparative genomics, Machine learning, Quantitative genetics, Genomic prediction, *Zea mays*

## Abstract

**Background:**

Crop improvement through cross-population genomic prediction and genome editing requires identification of causal variants at high resolution, within fewer than hundreds of base pairs. Most genetic mapping studies have generally lacked such resolution. In contrast, evolutionary approaches can detect genetic effects at high resolution, but they are limited by shifting selection, missing data, and low depth of multiple-sequence alignments. Here we use genomic annotations to accurately predict nucleotide conservation across angiosperms, as a proxy for fitness effect of mutations.

**Results:**

Using only sequence analysis, we annotate nonsynonymous mutations in 25,824 maize gene models, with information from bioinformatics and deep learning. Our predictions are validated by experimental information: within-species conservation, chromatin accessibility, and gene expression. According to gene ontology and pathway enrichment analyses, predicted nucleotide conservation points to genes in central carbon metabolism. Importantly, it improves genomic prediction for fitness-related traits such as grain yield, in elite maize panels, by stringent prioritization of fewer than 1% of single-site variants.

**Conclusions:**

Our results suggest that predicting nucleotide conservation across angiosperms may effectively prioritize sites most likely to impact fitness-related traits in crops, without being limited by shifting selection, missing data, and low depth of multiple-sequence alignments. Our approach—Prediction of mutation Impact by Calibrated Nucleotide Conservation (PICNC)—could be useful to select polymorphisms for accurate genomic prediction, and candidate mutations for efficient base editing. The trained PICNC models and predicted nucleotide conservation at protein-coding SNPs in maize are publicly available in CyVerse (10.25739/hybz-2957).

**Supplementary Information:**

The online version contains supplementary material available at 10.1186/s13059-022-02747-2.

## Background

In quantitative genetics, candidate causal mutations are often detected by statistical associations between genetic polymorphisms and phenotypic differences within species (QTL effects). QTL effects are useful in plant breeding (e.g., in genomic prediction), but they may be confounded by the co-segregation of neutral polymorphisms with causal mutations (linkage disequilibrium; LD) [1]. In contrast, phylogenetic nucleotide conservation (PNC) detects candidate causal mutations by conservation of DNA bases across species. This statistic is an indirect indicator of fitness effect [2], but it is less confounded by LD, due to the uncoupling of causal mutations and nearby polymorphisms, over large evolutionary timescales. PNC, as quantified by methods like SIFT [3] or gerp++ [4], may support plant breeding techniques which require identification of candidate causal mutations at high resolution: within hundreds of base pairs for cross-population genomic prediction or gene knock-out, and at single-base resolution for CRISPR-based editing.

Despite key advantages, PNC has practical disadvantages which limit its usefulness in quantitative genetics [5, 6]: (*i*) it is calculated from a multiple-sequence alignment (MSA), which requires cross-species conservation of alignable genomic regions; (*ii*) it may lack discriminatory power, because even variants with moderate effect on fitness may be highly conserved [2, 7]; and (*iii*) it may be biased by functional turnover (shifting selection) and clade-specific conservation. To overcome these limitations, PNC may be predicted throughout the genome, based on annotations which capture the genomic characteristics of fitness effects (genomic annotations). Previous methods like CADD [8, 9] and LINSIGHT [10, 11] have been introduced to predict PNC, using genomic annotations like epigenetic marks, amino acid change, or disruption of transcription factor motifs [9, 11]. However, they have relied on genomic annotations from large-scale experiments in human, which may not be available in plants. Moreover, the spatial resolution of their inference has been limited by small evolutionary timescales, within human and across related species.

In this study, we introduce a machine learning method to predict PNC across angiosperms in coding regions in maize (*Zea mays* L.), using computational annotations that are readily available from DNA sequence data and gene-model annotations. Computational annotations have several advantages: low cost, absence of missing values, and ease of portability from one genome to another. They may also provide latent (non-observed) representations of genes and can be used to perform in silico mutagenesis to predict the impact of point mutations on these representations. To achieve high resolution and high accuracy, we use high-resolution genomic annotations to predict PNC in the angiosperm clade, spanning > 140 million years of evolution and recombination events [12]. We use in silico mutagenesis to estimate the effect of mutations on protein structure, based on UniRep, a sequence-based deep learning technique which characterizes protein structure by latent representations of protein sequences [13]. Our predictions of PNC are validated by functional enrichment. Importantly, our validations include cross-population genomic prediction, in which genome-wide single-nucleotide polymorphisms (SNPs) are used to predict agronomic traits, and SNPs in coding regions are upweighted according to their predicted PNC. Together, our functional analyses show that predicted PNC is useful to identify impactful genes and SNPs for fitness-related traits in maize.

## Results

### Monomorphic sites in maize are under stronger evolutionary constraint than polymorphic sites

In this study, we aimed at capturing the genomic basis for fitness effects in coding regions in maize, by predicting PNC at nonsynonymous mutations from genomic annotations. PNC was estimated by conservation of DNA bases, in a MSA of 27 diverse plant genomes, from basal angiosperm *Amborella trichopoda*, to dicots (*Trifolium pratense*, *Medicago truncatula*, *Glycine max*, *Prunus persica*, *Populus trichocarpa*, *Manihot esculenta*, *Arabidopsis thaliana*, *Arabidopsis lyrata*, *Brassica napus*, *Brassica rapa*, *Theobroma cacao*, *Vitis vinifera*, *Solanum tuberosum*, *Solanum lycopersicum*, *Chenopodium quinoa*, *Beta vulgaris*) and monocots (*Sorghum bicolor*, *Setaria italica*, *Oryza rufipogon*, *Oryza longistaminata*, *Leersia perrieri*, *Triticum Urartu*, *Aegilops tauschii*, *Hordeum vulgare*, *Brachypodium distachyon*, *Musa acuminata*) [14]. Consequently, this MSA spanned large evolutionary times, equivalent to 16.2 expected substitutions per site under a neutral evolutionary model.

**Fig. 1:**
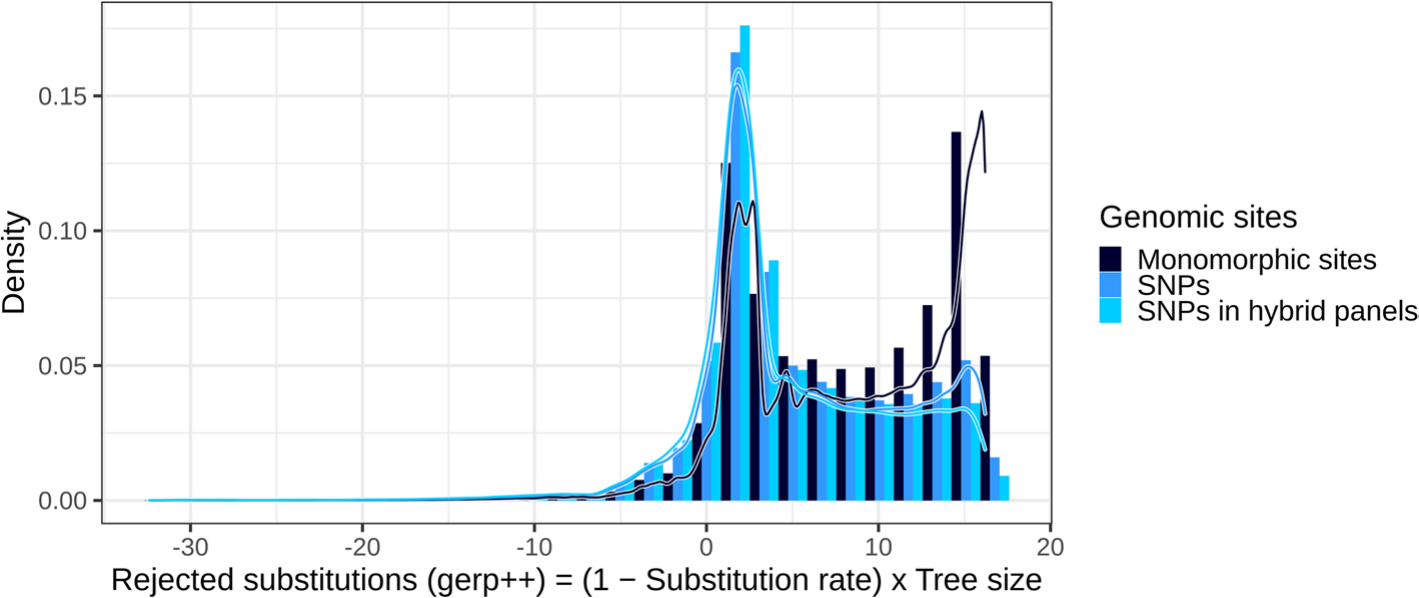
Distribution of rejected substitution (RS) scores by category of DNA bases. RS scores, which integrate information about conservation (1 − Substitution rate) and MSA depth (Tree size), were calculated by gerp++ [4] as previously described [14]. Monomorphic sites: sites with no observed polymorphism within maize. SNPs: observed polymorphisms in Hapmap 3.2.1, a representative panel of inbred lines in maize [15]. SNPs in hybrid panels: subset of SNPs which are observed in two panels of hybrid crosses between inbred lines and testers [16]

The DNA bases (genomic sites) with large fitness effects are subjected to evolutionary constraint, so they tend to be conserved across species (high PNC), and monomorphic within species (no observed polymorphism at the DNA base). Accordingly, monomorphic sites within maize (sites with no observed SNPs in a maize reference panel) tended to be more conserved across angiosperms, compared to polymorphic sites: they were aligned in MSAs over larger evolutionary timescales (Additional file 1: Fig. S1), and their evolutionary rate was lower (Fig. 1).

Our approach—Prediction of mutation Impact by Calibrated Nucleotide Conservation (PICNC)—used conserved sites as positive examples for large fitness effects, and sites in non-aligned regions as negative examples for neutral effects. PICNC did not rely on within-species variability (e.g., SNP allele frequency), so we could train our model on nonsynonymous mutations at any site, even when they were not observed in maize populations (i.e., even when they were monomorphic within maize). Therefore, we could include data about the genomic sites where polymorphisms are not tolerated by evolution (e.g., the sites where mutations are lethal). This helped us avoid survivorship bias at SNP sites and provided many more instances of PNC to learn about the genomic characteristics of fitness effects: 20,136,310 monomorphic sites, instead of 483,448 nonsynonymous SNPs across diverse maize lines [15] or 103,905 nonsynonymous SNPs in elite maize panels [16] (Fig. 1, Additional file 1: Fig. S1).

### Evolutionary constraint is accurately predicted by genomic annotations from sequence analysis

**Fig. 2:**
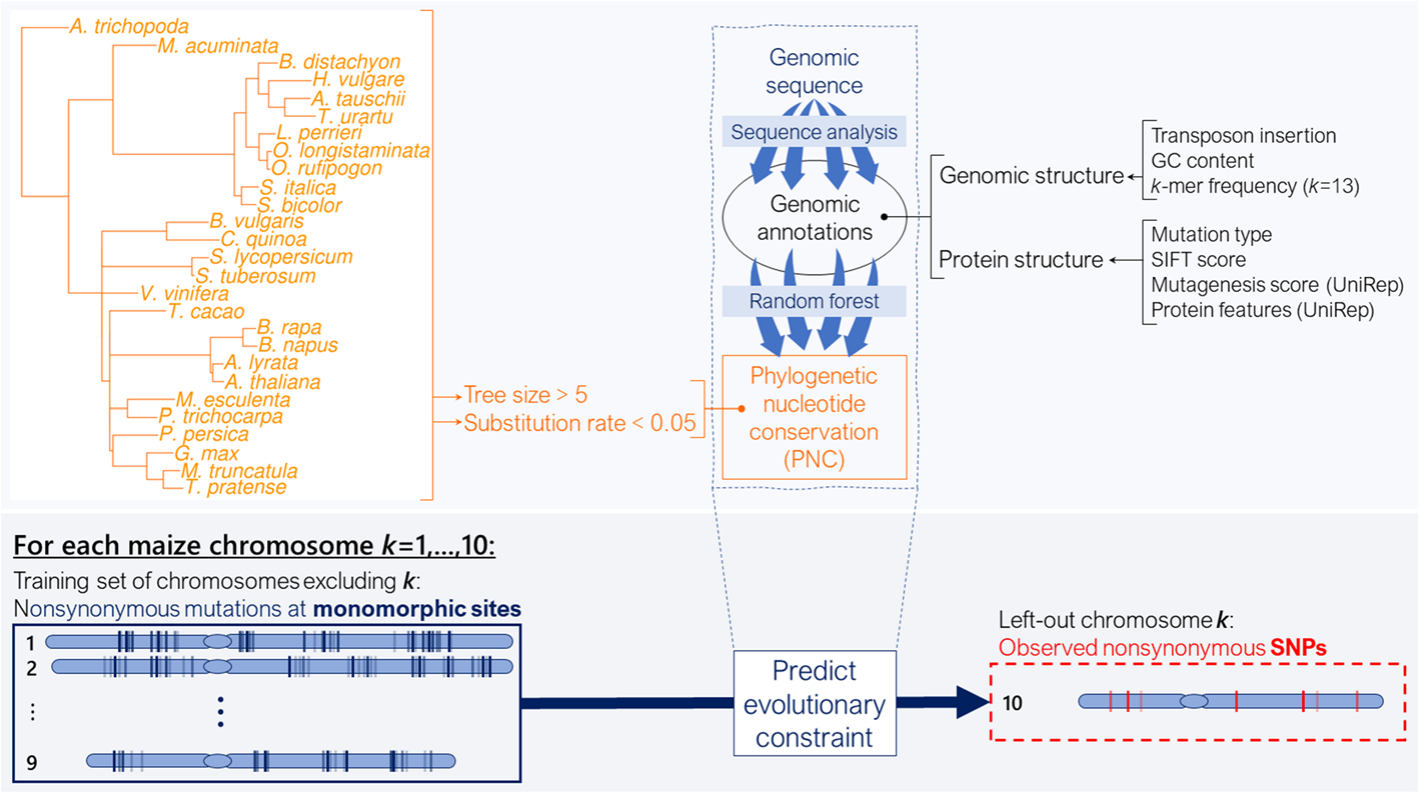
Prediction of mutation Impact by Calibrated Nucleotide Conservation (PICNC). Methodology for prediction of phylogenetic nucleotide conservation (PNC) by probability random forests. PNC was defined by high conservation (substitution rate < 0.05) over deep MSA (tree size > 5 expected neutral substitutions). Genomic annotations were produced only by sequence analysis. They described genomic structure and protein structure at nonsynonymous point mutations in maize coding regions. Monomorphic sites (no observed polymorphism within maize) were used for training, and observed SNPs were used for prediction. In leave-one-chromosome-out prediction, a probability random forest is trained ten times, once for each left-out chromosome

At each nonsynonymous mutation, PNC was characterized by a deep MSA (tree size > 5 expected nucleotide substitutions under a neutral model) and a high nucleotide conservation (substitution rate < 0.05 in the MSA at the site of the mutation). Observed PNC was used to train a probability random forest with genomic annotations about genomic structure (transposon insertion, GC content, average *k*-mer frequency) and protein structure (SIFT score, mutation type, protein features, and in silico mutagenesis scores from UniRep). While mutation type simply characterizes the codon change caused by a nonsynonymous mutation (missense, STOP gain, or STOP loss), SIFT score quantifies the impact of the nonsynonymous mutation by calculating the probability of its codon change (the lower the SIFT score, the more damaging the mutation) [3, 17]. In contrast, UniRep variables characterize the ontology and structure of a protein by a quantitative representation of its sequence [13]; in silico mutagenesis scores then quantify the effect of a mutation on this representation. Therefore, mutation type, SIFT score, and in silico mutagenesis scores all measured how damaging a codon change was, but in different ways. Our prediction approach (PICNC) benefited from three key advantages (Fig. 2): (*i*) monomorphic sites provided more information about PNC; (*ii*) annotations like SIFT scores and in silico mutagenesis scores from UniRep enabled predictions at single-site resolution; and (*iii*) leave-one-chromosome-out prediction avoided overfitting to observed PNC (see “Methods”). For each of the ten chromosomes in maize, we predicted PNC using a model trained in all other chromosomes. Therefore, the accuracy of our approach could not be inflated by spurious associations between genomic annotations and PNC along chromosomes. For each left-out chromosome, the PICNC random forest model was tuned for optimal hyperparameters (number of trees per forest, and number of sampled features per tree; see “Methods”). Our model showed little sensitivity to hyperparameters, as exemplified by the low range of classification accuracy in chromosome 8 (0.6%) across hyperparameters (Additional file 1: Fig. S2).

**Fig. 3:**
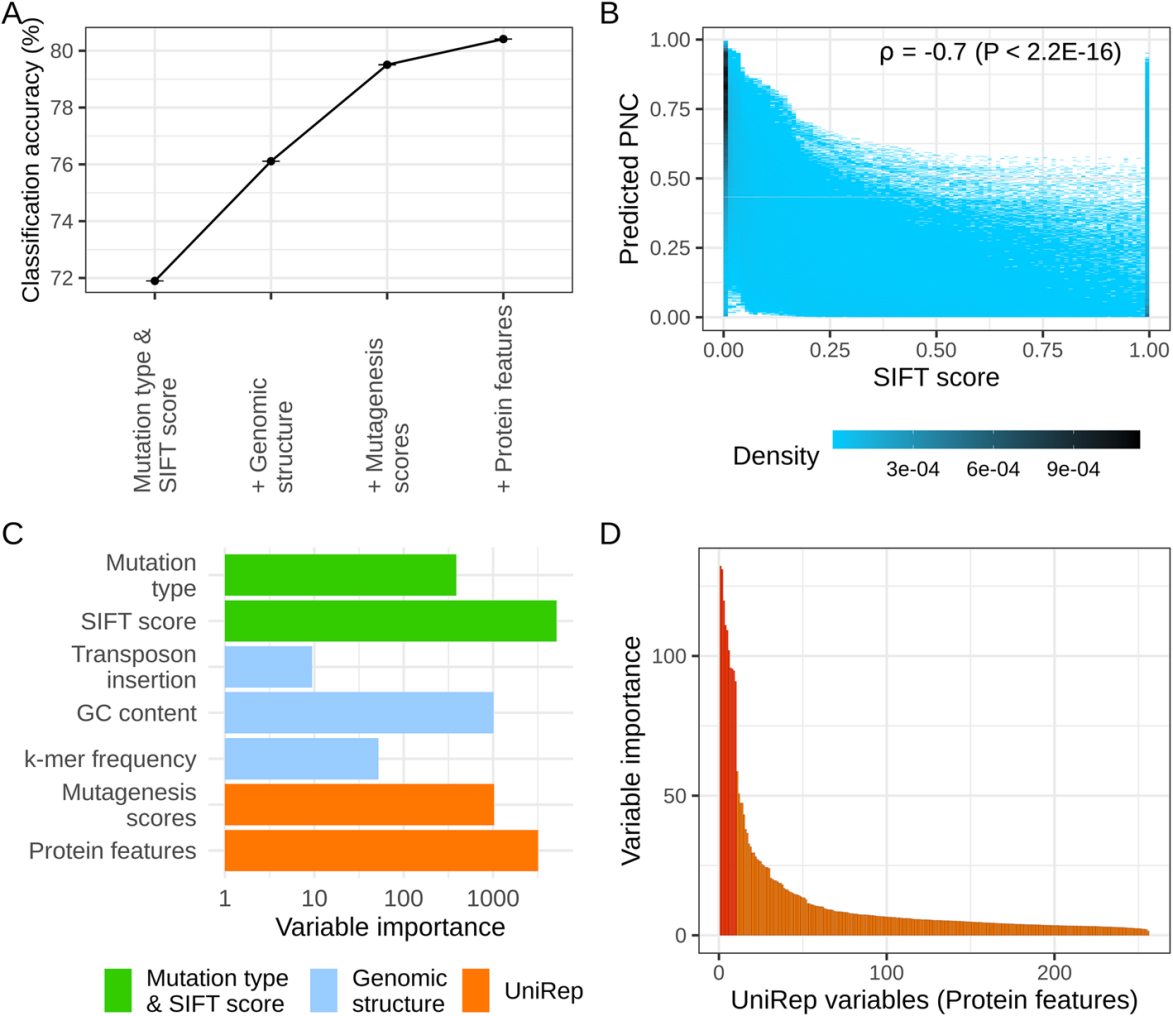
Contribution of genomic annotations to prediction accuracy in probability random forests. **A** Classification accuracy of probability random forests for predicted phylogenetic nucleotide conservation (PNC). Accuracy: percentage of correct calls, i.e., the percentage of sites in chromosome 8 for which predicted PNC (rounded) equaled observed PNC, over three replicates. Accuracy was weighted to account for imbalance with respect to PNC (see “Methods”). Sets of genomic annotations were sequentially added to the set of predictors in probability random forests. Mutation type & SIFT score: Mutation type (missense, STOP gain or STOP loss), SIFT score (with missing values set to 1), and SIFT class (“constrained” if SIFT score ≤ 0.05, “tolerated” otherwise). Genomic structure: GC content, *k*-mer frequency and transposon insertion. Mutagenesis scores: in silico mutagenesis scores for UniRep variables. Protein features: UniRep variables, generated by the 256-unit UniRep model. **B** Relationship between SIFT scores and predicted PNC at maize SNPs (observed polymorphisms in Hapmap 3.2.1, a representative panel of inbred lines in maize [15]). Predicted PNC is computed by the full PICNC model, including all genomic annotations. Darker colors indicate higher density of SNPs. *ρ*: Spearman correlation coefficient. **C** Variable importance of genomic annotations. Variable importance: corrected impurity measure in probability random forests [18]. **D** Variable importance of protein features (UniRep variables), ordered in decreasing order. A subset of 10 UniRep variables stood out as contributing most to the prediction accuracy for PNC

Compared to a baseline model including SIFT score and mutation type (missense, STOP gain, STOP loss), annotations about genomic structure (especially GC content) contributed to an improved prediction accuracy for PNC, from 72 to 76% (Fig. 3A). Protein features (UniRep variables) and their in silico mutagenesis scores resulted in a further increase to > 80% (Fig. 3A). This additional gain in accuracy suggests that novel annotations about protein structure and the impact of nonsynonymous mutations, based on machine learning techniques (protein embedding) rather than evolutionary or bioinformatic approaches, may improve our ability to detect deleterious mutations in protein-coding regions. Predicted PNC was correlated to SIFT scores, such that sites with a minimum SIFT score (0) tended to have a large predicted PNC (Fig. 3B). However, this concordance was not perfect, and predicted PNC pointed to potential false positives: sites under low evolutionary constraint among those with a minimum SIFT score. As expected, SIFT score was the most useful genomic annotation for predicting PNC, but its importance was on par with those of UniRep variables and their in silico mutagenesis scores (Fig. 3C). The importance of these annotations suggests that information about protein structure may have enabled finer prioritizations, compared to a baseline model which only included mutation type and SIFT score.

To investigate the usefulness of UniRep variables within maize, we fitted random forests models which regressed gene properties on UniRep variables: expression levels (RNA and protein abundance) and selective constraint (negatively associated with the nonsynonymous-to-synonymous SNP ratio, *P*_n_/*P*_s_, and the proportion of nonsynonymous SNPs, *P*_n_/(*P*_n_+*P*_s_), within each gene). UniRep variables captured gene variability within maize, for these gene properties: prediction accuracy (Pearson correlation) > 0.35 (Additional file 1: Fig. S3). Therefore, the UniRep variables, which were designed to capture protein structural variability across viruses, prokaryotes, and eukaryotes, were useful, both across angiosperms (on PNC) and within maize (on gene properties). Interestingly, a subset of 10 variables stood out as capturing more information about PNC (Fig. 3D) and was also important for predicting selective constraint within species, as reflected by the nonsynonymous-to-synonymous SNP ratio and the proportion of nonsynonymous SNPs within genes (Additional file 1: Fig. S4) [19]. Therefore, few UniRep variables may capture the fitness effects of maize genes and could serve as succinct functional representations of genes for effects on fitness-related traits.

UniRep variables improved classification accuracy for PNC in protein-coding regions. However, such an improvement was not observed for mutations in non-coding regions of genes (introns and UTRs) or intergenic regions, when using UniRep variables to describe protein features of the nearest gene (Additional file 1: Fig. S5). Therefore, while UniRep variables may be useful to predict the effects of mutations on protein structure, they may not provide useful information for effects on other biological processes (e.g., regulation of gene expression).

### Predicted evolutionary constraint identifies deleterious variants

**Fig. 4:**
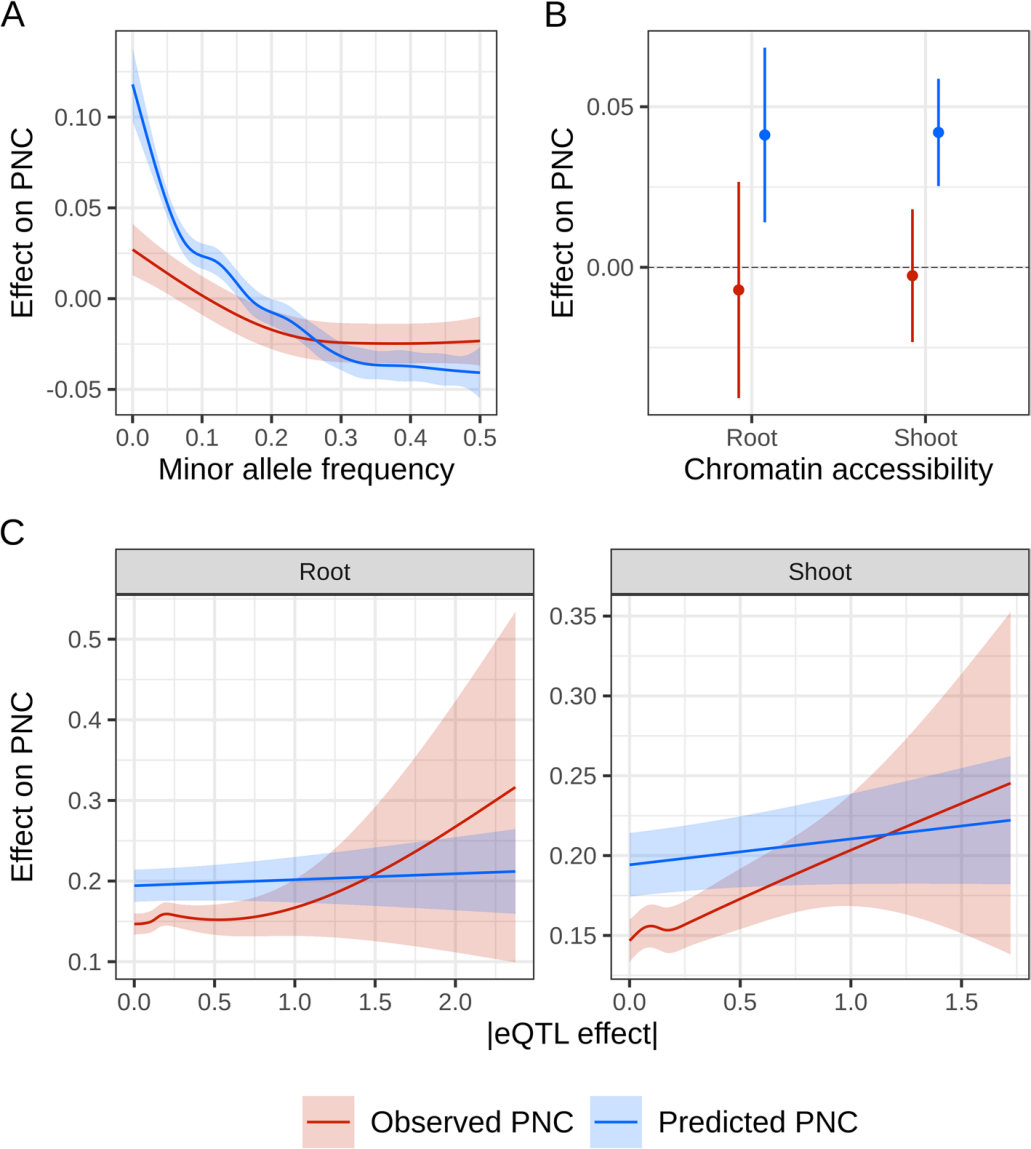
Relationship between phylogenetic nucleotide conservation (PNC) and experimental annotations at SNPs. **A** Decrease in observed and predicted PNC over within-species variability, quantified by MAF in reference panel Hapmap 3.2.1 [15]. **B** Increase in predicted PNC in accessible chromatin regions, defined by MNase hypersensitivity in shoot or root tissues [21]. **C** Positive association between observed PNC and expression QTL effect (in absolute values) in shoot or root tissues, estimated in a diverse maize panel [22]

**Fig. 5:**
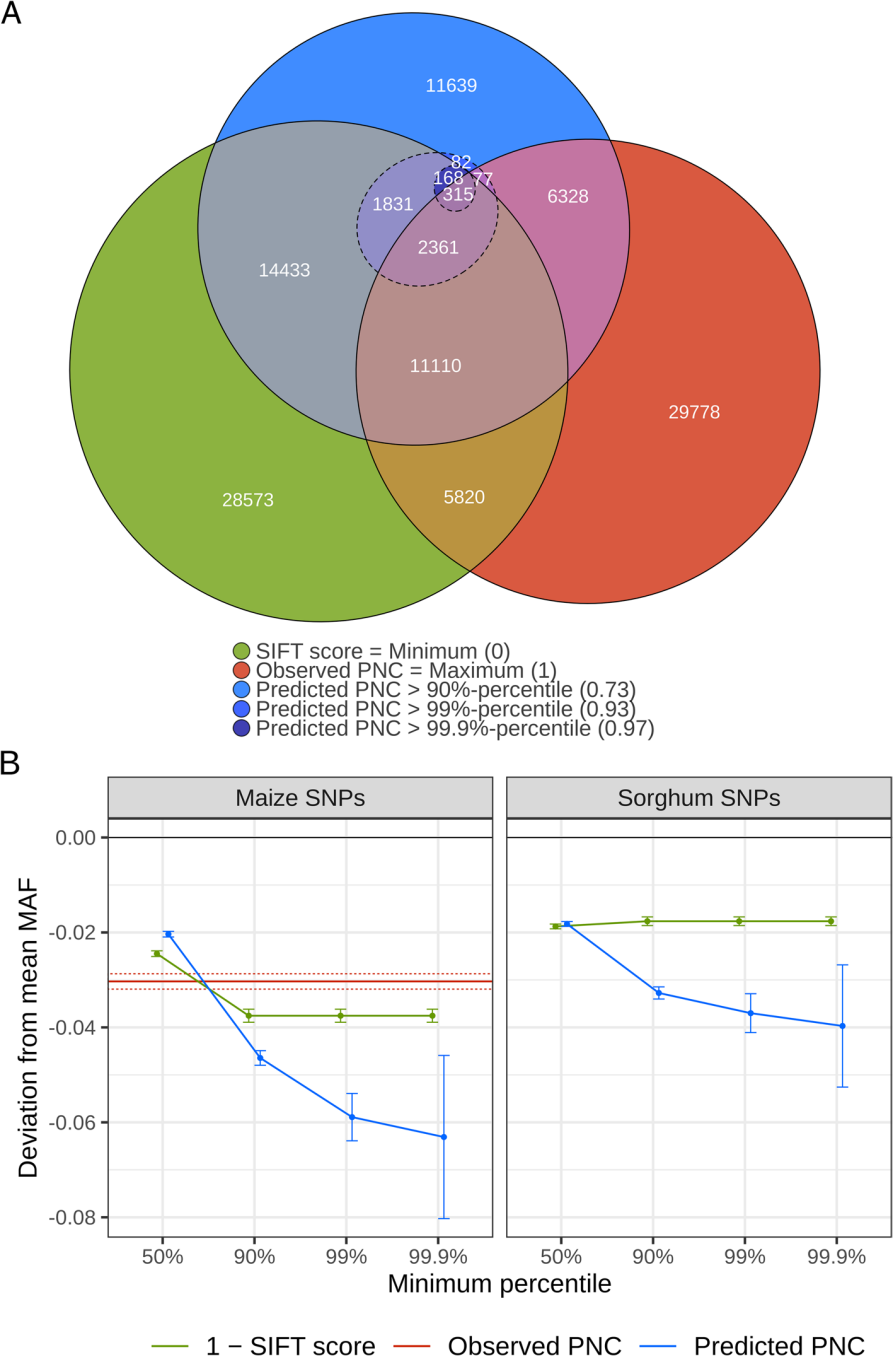
Prioritization of SNPs by SIFT score or phylogenetic nucleotide conservation (PNC). **A** Euler diagram of SNP sets prioritized by SIFT score = 0, observed PNC = 1, or predicted PNC > 90%, 99%, 99.9% percentile, in maize (observed polymorphisms in Hapmap 3.2.1). Concentric dashed circles refer to smaller subsets of SNPs prioritized by increasingly stringent thresholds on predicted PNC. **B** Decrease in minor allele frequency (MAF) of prioritized SNPs, in maize and sorghum. Difference in MAF between prioritized SNPs and all SNPs. Maize SNPs: observed polymorphisms in the Hapmap 3.2.1 reference panel [15]; Sorghum SNPs: observed polymorphisms in the reference panel of Lozano et al. [23]. SNPs were prioritized if their SIFT conservation (1 − SIFT score) or predicted PNC was above the 50%, 90%, 99%, or 99.9% percentile, or if their observed PNC was equal to 1 (tree size > 5, substitution rate < 0.05). Error bars and dotted lines represent 95% confidence intervals in two-sample *t*-tests, for SIFT score or predicted PNC, and observed PNC, respectively

Observed PNC is prone to errors and lacks power to discriminate among different sizes of fitness effects [6]. On the other hand, predicted PNC is estimated by functions of genomic annotations learned across many sites and provides a quantitative value for the probability of PNC, ranging from 0 to 1. Therefore, we tested the hypothesis that predicted PNC could estimate fitness effects more accurately than observed PNC. Variability at SNPs, as reflected by minor allele frequency in a maize reference panel (MAF), provided information about selective constraint at DNA sites within species. The relationship between PNC and fitness effects was corroborated by its negative association with MAF, as was previously reported [20]. Notably, SNPs prioritized by predicted PNC tended to have lower MAF as prioritizations grew more stringent, and these SNPs were eventually much rarer than those prioritized by observed PNC (Figs. 4A and 5B). The functional relevance of predicted PNC was also supported by its positive association with chromatin accessibility (Fig. 4B), which is correlated with phenotypic effects in maize [16, 21]. A positive association with expression QTL (eQTL) effect was also observed, but only for observed PNC (*P* = 0.003 and *P* = 0.034 in shoot and root tissues respectively, compared to *P* = 0.120 and *P* = 0.485 for predicted PNC; Fig. 4C), possibly because the genomic annotations used to predict PNC lack relevant information about gene expression, or because the sites in eQTL are not under strong negative selection across angiosperms.

SIFT score (the more conserved the site, the lower) and predicted PNC (the more conserved, the higher) are both proxies for evolutionary constraint. However, SIFT scores pointed to relatively large sets of SNPs in the maize reference panel; even the smallest SIFT score value pointed to as many as 64,611 out of 483,448 nonsynonymous SNPs in a maize reference panel (Fig. 5A). Predicted PNC above its 90% percentile (0.73) pointed to 48,345 SNPs, most of which were also prioritized by SIFT score or observed PNC. However, more stringent thresholds on predicted PNC pointed to smaller subset of SNPs, within the SNP set prioritized by minimum SIFT score (Fig. 5A). Under the hypothesis that predicted PNC identifies signatures of negative selection, we expected a decrease in average MAF as SNP prioritizations grew more stringent, even for the relatively small subsets selected by predicted PNC above the 99% and 99.9% percentiles. As expected, predicted PNC achieved a decrease in average MAF in a maize reference panel [15], which was larger as SNP prioritizations were more stringent, and was significantly larger than the decrease achieved by either observed PNC or SIFT score (Fig. 5B). We also estimated the average minor allele frequency in a sorghum reference panel [23], and we predicted PNC in the sorghum reference genome, using a PICNC model trained on all maize chromosomes (using all genomic annotations except transposon insertion; see “Methods”). As expected, SNP prioritizations by predicted PNC also resulted in a decrease in minor allele frequency in sorghum, which was significantly larger compared to prioritizations by SIFT score (Fig. 5B).

### Predicted evolutionary constraint prioritizes highly expressed genes in primary metabolic pathways

**Fig. 6:**
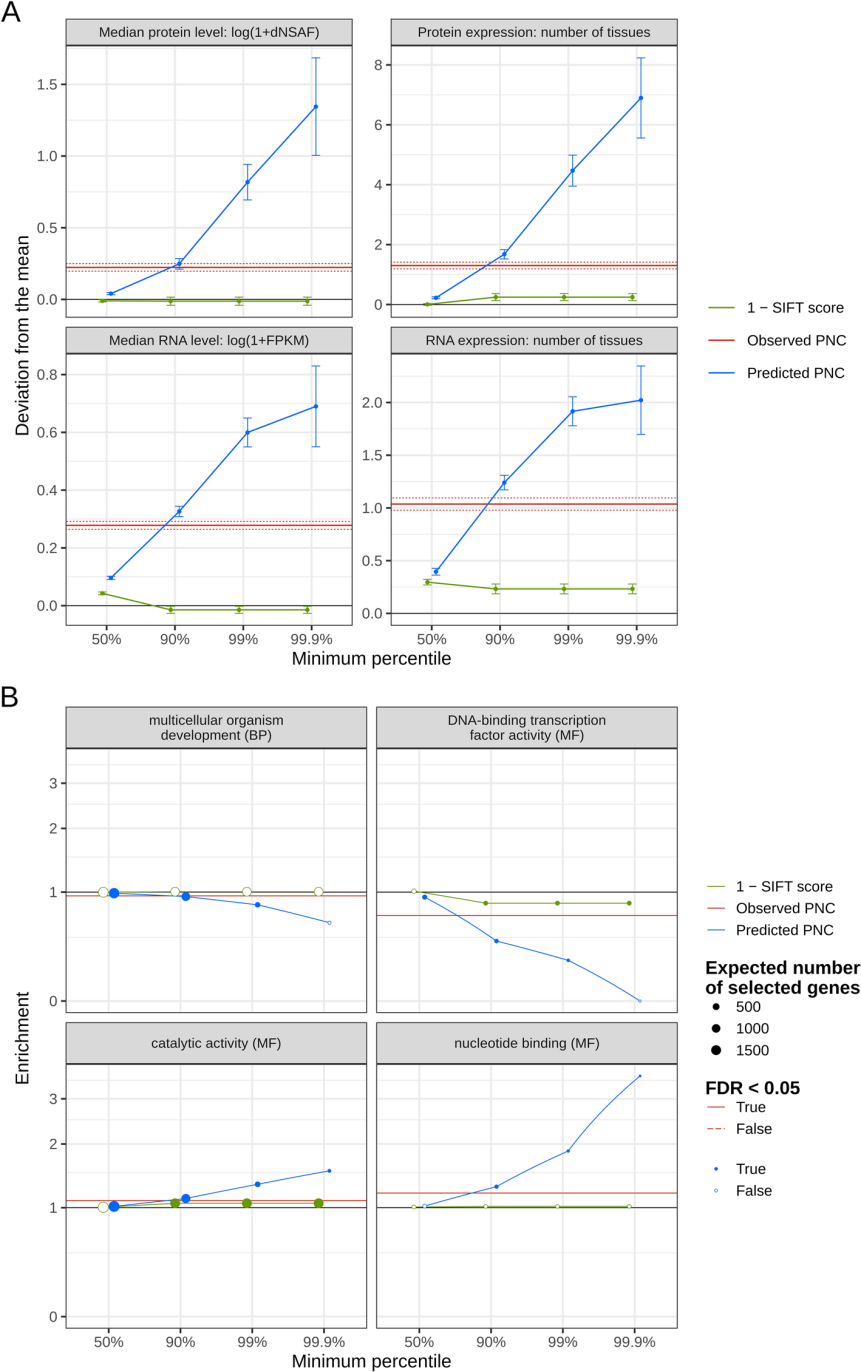
Functional enrichment of genes prioritized by SIFT score or phylogenetic nucleotide conservation (PNC), in maize. Genes were prioritized by selecting SNPs with SIFT conservation (1 − SIFT score) or predicted PNC above the 50%, 90%, 99%, or 99.9% percentile, or observed PNC equal to 1 (tree size > 5, substitution rate < 0.05). **A** Difference in average expression between prioritized genes and all genes. Gene expression is quantified by RNA abundance (FPKM over 23 tissues) and protein abundance (dNSAF over 32 tissues) based on the gene expression atlas of [29]: median expression, and number of tissues with non-zero expression level. Error bars and dotted lines represent 95% confidence intervals in two-sample *t*-tests, for predicted and observed PNC, respectively. **B** Enrichment of prioritized genes for gene ontology (GO) classes. Ratio of number of prioritized genes over expected number under the null hypothesis (random gene prioritization). GO classes belong to the plant GO slim subset [30]. Ontology: BP, biological process; MF: molecular function. For each threshold and ontology, false discovery rates (FDR) were calculated over GO classes, based on *P*-values from Fisher’s exact tests. Full circles and full lines indicate FDR < 0.05, for SIFT conservation or predicted PNC, and observed PNC, respectively

Under the hypothesis that predicted PNC identifies impactful genes, the set of genes prioritized by predicted PNC should be enriched for important functional attributes like high gene expression. Prioritization by SIFT score (17,101 or more genes selected) resulted in a small increase in protein expression, while observed PNC resulted in significant enrichment for highly expressed genes (higher RNA and protein abundance, in more tissues), among 14,646 prioritized genes out of the 24,549 genes containing nonsynonymous SNPs (Fig. 6A). However, such enrichment was more evident with predicted PNC, and increased consistently as fewer genes were selected (Fig. 6A). As expected, the nonsynonymous-to-synonymous SNP ratio and the proportion of nonsynonymous SNPs within genes also decreased as prioritization of genes grew more stringent, in maize as well as in sorghum (Additional file 1: Fig. S6). These results suggest that predicted PNC pointed to impactful genes. Alternatively, PNC at these prioritized genes may be a direct consequence of “expression-rate anticorrelation,” i.e., selection against cytotoxic byproducts of highly expressed genes (e.g., due to mRNA misfolding or protein misinteraction), rather than direct selection for their functional importance [24–28].

To analyze the function of genes prioritized by predicted PNC, we estimated their enrichment for GO classes. Significant enrichment was detected for genes involved in catalytic activity and nucleotide binding (e.g., ATP binding for energy transfer). Based on these functional enrichments, predicted PNC prioritized genes involved in primary metabolism (Fig. 6B, Additional file 1: Fig. S7). In contrast, genes involved in gene regulation and plant development were depleted by these prioritizations. Prioritization by observed PNC also resulted in significant depletion for these GO classes, so PNC across angiosperms may have de-emphasized developmental genes, possibly because of functional turnover over large evolutionary timescales [5, 6]. Even though we included PNC over moderate evolutionary timescales (tree size between 5 and its maximum, 16.2), clade-specific constraint (e.g., at the genus level) could not be detected in the sample of genomes used in this study [14]. In addition, the depletion by predicted PNC may have been exacerbated by the prediction model itself; the absence of genomic annotations about gene regulation (e.g., RNA-protein binding) may have downplayed the importance of developmental genes for fitness. Finally, these depletions might actually reflect relaxed selection on low gene expression (expression-rate anticorrelation) [28]. However, even after accounting for RNA and protein expression, we still observed significant depletions for these GO classes (Additional file 1: Fig. S7), so we could not rule out genes’ functional importance as a direct determinant of PNC.

For a more detailed description of prioritized genes, we identified the pathways that were significantly enriched among the genes prioritized by predicted PNC, observed PNC, or SIFT score. According to the CornCyc database [31], gene prioritization by SIFT score or observed PNC did not result in significant pathway enrichment, while predicted PNC pointed to genes involved in carbon metabolism (Table 1). The most represented pathways among genes prioritized by predicted PNC (> 99.9% percentile) were in “C4 photosynthetic carbon assimilation cycle” (Additional file 2: Tables S1 and S2), while significant enrichment was detected for pathways involved in glycolysis, fatty acid catabolism, and amino acid biosynthesis (Table 1). Therefore, pathway enrichments confirmed GO enrichments and pointed to carbon-metabolic pathways likely shared across angiosperms.

**Table 1:** Enrichment of prioritized genes for CornCyc pathway annotations

PNC	Minimum percentile	Pathway	Enrichment	***P***-value (FDR)
**Observed**	NA	Triacylglycerol degradation (LIPAS-PWY)	0.76	2.9 × 10^−5^ (0.0093)
		Aerobic respiration III (alternative oxidase pathway) (PWY-4302)	0.70	3.7 × 10^−5^ (0.0093)
**Predicted**	90%	Glycolysis IV (plant cytosol) (PWY-1042)	1.41	7.8 × 10^−7^ (0.00038)
		Rubisco shunt (PWY-5723)	1.40	2.1 × 10^−4^ (0.015)
		Glycolysis I (from glucose 6-phosphate) (GLYCOLYSIS)	1.36	1.0 × 10^−5^ (0.0025)
		Gluconeogenesis I (GLUCONEO-PWY)	1.36	8.4 × 10^−5^ (0.0083)
		Glycolysis II (from fructose 6-phosphate) (PWY-5484)	1.36	2.4 × 10^−5^ (0.0029)
		Triacylglycerol degradation (LIPAS-PWY)	0.65	1.7 × 10^−5^ (0.0027)
		Very long-chain fatty acid biosynthesis I (PWY-5080)	0.59	3.1 × 10^−4^ (0.019)
		*Trans*-zeatin biosynthesis (PWY-2681)	0.29	1.6 × 10^−4^ (0.013)
	99%	L-leucine biosynthesis (LEUSYN-PWY)	3.66	9.5 × 10^−4^ (0.043)
		Fatty acid β-oxidation II (peroxisome) (PWY-5136)	3.59	4.8 × 10^−4^ (0.033)
		Glyoxylate cycle (GLYOXYLATE-BYPASS)	2.99	6.3 × 10^−4^ (0.033)
		TCA cycle II (PWY-5690)	2.51	5.0 × 10^−4^ (0.033)
		Gluconeogenesis I (GLUCONEO-PWY)	2.24	2.6 × 10^−4^ (0.032)
		Glycolysis IV (plant cytosol) (PWY-1042)	2.16	2.0 × 10^−4^ (0.032)
		Glycolysis II (from fructose 6-phosphate) (PWY-5484)	2.16	2.0 × 10^−4^ (0.032)
		Glycolysis I (from glucose 6-phosphate) (GLYCOLYSIS)	2.09	4.4 × 10^-4^ (0.033)
		Aerobic respiration I (cytochrome c) (PWY-3781)	0.31	4.3 × 10^−6^ (0.0021)
		Triacylglycerol degradation (LIPAS-PWY)	0.23	6.5 × 10^−4^ (0.033)
		Very long-chain fatty acid biosynthesis I (PWY-5080)	0	6.6 × 10^−4^ (0.033)

PNC: Measure of phylogenetic nucleotide conservation (PNC) used to select SNPs in the maize reference panel (Hapmap 3.2.1) and prioritize the genes containing the selected SNPs; SNPs were selected if their observed PNC was 1 or if their predicted PNC was above the 50%, 90%, 99%, or 99.9% percentile; Pathway: name and ID of pathway, retrieved from CornCyc, release 2021/03/25 [31]; Enrichment: ratio of observed over expected counts; *P*-value, from Fisher’s exact test; FDR, false discovery rate [32] to correct for multiple testing over pathways. Prioritization of genes by SIFT scores did not result in any statistically significant enrichment or depletion for pathway annotations

### Predicted evolutionary constraint improves genomic prediction for fitness-related traits in hybrid maize lines

**Fig. 7:**
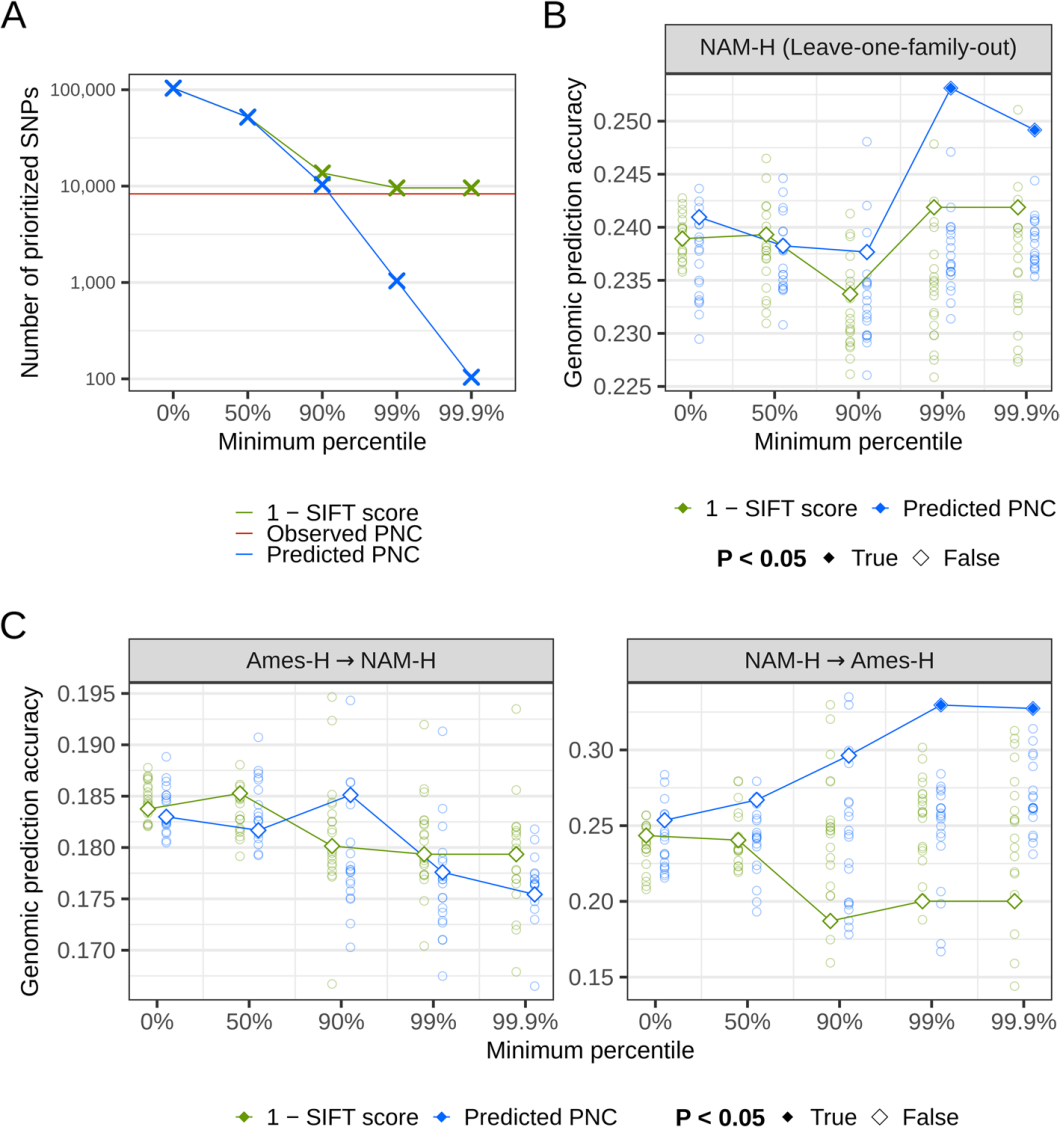
Prioritization of nonsynonymous SNPs in genomic prediction for grain yield, in hybrid maize lines. **A** Number of SNPs prioritized by SIFT conservation (1 − SIFT score), predicted phylogenetic nucleotide conservation (PNC), or observed PNC. **B** Genomic prediction accuracy within panel, in leave-one-family-out prediction in the Nested Association Mapping hybrid panel (NAM-H) [16]. **C** Genomic prediction accuracy across panels, from a diverse hybrid panel (Ames-H) to NAM-H, and vice versa [16]. Genomic prediction models included effects of population structure variables (top three principal components in the Hapmap 3.2.1 reference panel in maize), effects of genome-wide SNPs, and effects of nonsynonymous SNPs. Diamonds: nonsynonymous SNPs were weighted by SIFT conservation or predicted PNC, and prioritized by truncating weights to zero if they were under the 0%, 50%, 90%, 99%, or 99.9% percentile. Open circles: nonsynonymous SNPs were weighted and prioritized by 20 random permutations of SIFT conservation or predicted PNC, to determine whether the prediction accuracy by SNP weights was significantly different from the accuracy by random SNP weights

To assess the functional relevance and practical utility of predicted PNC, we used predicted PNC to weight nonsynonymous SNPs in genomic prediction for agronomic traits: days to silking (DTS), plant height (PH), or grain yield (GY). We tested the hypothesis that predicted PNC was larger at causal variants for fitness-related traits in hybrid panels. Under this hypothesis, we expected that (i) weighting SNPs with predicted PNC increased the accuracy of genomic prediction; and (ii) prioritizing SNPs with larger predicted PNC resulted in further gains in accuracy. Expectation (i) was not met for any of the agronomic traits (Additional file 1: Fig. S8), probably because of the large LD extent in the hybrid panels (average squared correlation above 0.1 within 100-kb distance), such that causal variants were adequately tagged even by randomly weighted SNPs [16]. Expectation (ii) was met for GY, our trait most related to fitness; a gradual increase in prediction accuracy was observed as prioritization of SNPs was more stringent (Fig. 7), with a trend similar to that for lower MAF (Fig. 5B). Moreover, a significant increase in prediction accuracy was obtained by prioritizing the top 1040 (1%) and 104 (0.1%) SNPs (*P* < 0.05 based on random permutations of SNP weights), while prioritization by the lowest SIFT score (9576 SNPs) did not result in a significant increase in prediction accuracy (Table 2, Fig. 7). The gains in prediction accuracy achieved by predicted PNC were greater than those achieved by observed PNC (Table 2), despite ~80 times fewer prioritized SNPs (Fig. 7A, Additional file 2: Table S3). Assuming the minor allele to be deleterious, prioritization of the top 0.1% SNPs by predicted PNC would select 15 mutations per inbred line on average (average MAF of 0.144 among prioritized SNPs; Additional file 2: Table S3). Therefore, stringent prioritization of SNPs by predicted PNC could enable the selection of manageable numbers of candidate variants, for subsequent purging of mutational load by breeding or CRISPR-based editing. Such few selected variants would represent a small fraction of the total mutational load, since even most stringent prioritizations by SIFT scores would select as many as 1638 deleterious mutations per inbred line on average (average MAF of 0.171 among prioritized SNPs; Additional file 2: Table S3). However, they would represent the fraction of most impactful SNPs in each haploid genome.

**Table 2:** Genomic prediction accuracy in hybrid maize lines by prioritization of nonsynonymous SNPs in coding regions

				SNP prioritization										
				Baseline	Observed PNC	Predicted PNC					SIFT conservation (1 − SIFT score)			
**Minimum percentile**				None	None	0%	50%	90%	99%	99.9%	0%	50%	90%	99%
**Validation**	Ames-H ➔ NAM-H	**Trait**	DTS	0.775	0.773	0.776	0.777	0.775	0.773	0.771^a^	0.776^a^	0.777^a^	0.775	0.774
			PH	0.365	0.367	0.367	0.368	0.358	0.367	0.371	0.364	0.365	0.376	0.369
			GY	0.185	0.179	0.183	0.182	0.185	0.178	0.175	0.184	0.185	0.180	0.179
	NAM-H ➔ Ames-H		DTS	0.504	0.500	0.503	0.503	0.501	0.491	0.496	0.501	0.508	0.459^a^	0.454^a^
			PH	0.231	0.036	0.199	0.220	0.168	0.276^a^	0.111	0.182	0.137	0.079^a^	0.148
			GY	0.240	0.307^a^	0.254	0.267	0.296	0.330^a^	0.327^a^	0.243	0.240	0.187	0.200
	NAM-H (Leave-one-family-out)		DTS	0.404	0.405	0.402	0.401^a^	0.402	0.403	0.403	0.403	0.403	0.402	0.403
			PH	0.397	0.388	0.395	0.393	0.393	0.397	0.392	0.396	0.397	0.392	0.392
			GY	0.240	0.231	0.241	0.238	0.238	0.253^a^	0.249^a^	0.239	0.239	0.234	0.242

Genomic prediction models included effects of population structure variables (top three principal components from the Hapmap 3.2.1 reference panel in maize), genome-wide SNP effects, and prioritized effects of nonsynonymous SNPs. SNP prioritization consisted of weighting nonsynonymous SNPs uniformly (Baseline), or by a proxy for evolutionary constraint: observed phylogenetic nucleotide conservation (PNC), predicted PNC, or SIFT conservation (1 − SIFT score). For SNP prioritization by predicted PNC and SIFT conservation, SNP weights were also truncated to zero below their 0%, 50%, 90%, 99%, or 99.9% percentile. Validation: Ames-H ➔ NAM-H, training in a diverse hybrid panel (Ames-H) and validation in the Nested Association Mapping hybrid panel (NAM-H); NAM-H ➔ Ames-H, training in NAM-H and validation in Ames-H; NAM-H (leave-one-family-out), validation in each family in NAM-H after training in all other families. Trait: *DTS* days to silking, *PH* plant height, *GY* grain yield

^a^Prediction accuracy was significant at the 5% level, based on random permutation of SNP weights. Underlined values indicate significant improvements over the baseline

Significant increase in prediction accuracy for GY was observed in a large panel of half-sibs (NAM-H), but not in a diverse panel representative of genetic variability in maize (Ames-H). Importantly, SNPs that are rare in maize are not necessarily rare in NAM-H, because of the half-sib family structure, in which 24 different donor parents are crossed to the same recurrent parent [16]. Therefore, effects of deleterious mutations from the recurrent parent in NAM-H were estimated accurately, even though they may be rare in maize. The gain in prediction accuracy achieved by predicted PNC was significant but modest (0.25 by prioritizing the top 0.1% vs. 0.24 by weighting all nonsynonymous SNPs equally), probably because the donor parents were unrelated and shared few deleterious mutations with one another (Table 2, Fig. 7B). However, when we used NAM-H to predict GY in Ames-H, we achieved a large and significant increase in prediction accuracy (0.33 by prioritizing the top 0.1% vs. 0.24 with equal weights; Table 2, Fig. 7C). In Ames-H, variation at SNPs—and the information available to learn their effect—is positively correlated with MAF [16]. Therefore, prioritization of variants with lower MAF (Figs. 4A and 5B) resulted in larger estimation errors in Ames-H and may explain why genomic prediction models trained in this panel benefited less from prioritizations by predicted PNC (Fig. 7C, Additional file 1: Fig. S8). Accordingly, genomic effects at rare SNPs (MAF < 0.01) were significantly larger in NAM-H (> 16-fold enrichment) but not in Ames-H, based on a previous study [16]. Therefore, NAM-H was a useful training set to test enrichments of prioritized SNPs for genomic effects, but Ames-H did not allow us to detect such enrichment due to the relatively low variability at rare SNPs in this panel.

Genomic prediction for other agronomic traits (PH and DTS) was not improved by PNC. This lack of improvement may be due to a weak relationship between these traits and evolutionary constraint, as proxied by PNC across angiosperms. Consistently, in maize, hybrid vigor and inbreeding depression are substantially larger for traits related to seed weight and grain yield, compared to traits related to plant morphology and flowering time [33–35]. Interestingly, prioritizations by predicted PNC resulted in a gradual decrease and a significant loss of accuracy for DTS, in a genomic prediction model trained in Ames-H, which suggests that predicted PNC may actually fail to detect variants that are causal for adaptive traits like flowering time (Table 2, Additional file 1: Fig. S8). Moreover, enrichment of rare SNPs (MAF < 0.01) for effects on PH and DTS was not detected in either Ames-H or NAM-H [16], which suggests that the SNPs impacting these traits are under weaker negative selection, compared to those impacting GY. Together, our results on PNC, and previous results on MAF, indicate that prioritization of SNPs by PNC may improve genomic prediction if some of their causal SNPs are under negative selection, and carry enough statistical information in the training set (e.g., causal SNPs for a yield trait like GY in a collection of biparental populations like NAM-H).

## Discussion

Our results about the characteristics of prioritized SNPs and genes suggest that predicted PNC is more useful than observed PNC to identify causal variants for fitness-related traits, since it can select fewer variants and produce stronger functional enrichments. Our approach (PICNC) addressed two important caveats of observed PNC, which limit its usefulness for quantitative genetics and breeding applications: missing information outside MSAs, and sensitivity of conservation to fitness effects [6]. Our predictions of PNC addressed the first caveat by using, as predictors, genomic annotations that are readily available from DNA sequence data and gene-model annotations. These genomic annotations were produced by bioinformatic and machine learning procedures which are designed for broad sets of species, with the exception of transposon insertion which was detected by maize-specific transposon motifs [36] but was not important in our predictions (Fig. 3C). The second caveat is due to low discriminatory power of observed PNC, such that PNC tends to its maximum as soon as evolutionary constraint is moderate, especially in MSA across few taxa [2, 7]. Our predictions addressed this caveat by estimating a probability for PNC, which could be used to select arbitrarily small sets of sites in prioritizations (Figs. 5A and 7A), whereas observed PNC might select too many sites for breeding applications like biological design (e.g., 55,789 and 8311 nonsynonymous SNPs with observed PNC in reference and hybrid panels, respectively).

Based on GO and pathway enrichment analyses, predicted PNC pointed to genes in primary metabolism: biosynthesis and energetic catabolism (Fig. 6B, Table 1). However, genes involved in secondary (specialized) metabolism were not preferentially selected, despite their importance in adaption (e.g., resistance to biotic or abiotic stress). The prioritization of impactful variants in such genes will require a proxy for fitness effects that is specific to environmental conditions: cultivated or wild environment, and clade-specific selection pressures [37]. The detection of fitness effects acting through secondary metabolism will certainly require to predict PNC over smaller evolutionary timescales (e.g., within the Andropogoneae clade) [38], by genomic annotations that are specific to tissues and/or environments [39]. The emphasis of predicted PNC on primary metabolism illustrates a fundamental trade-off in our approach (and other similar approaches like SIFT): on the one hand, evolutionary depth allows us to detect effects of mutations at high spatial resolution; on the other hand, inferences are biased towards certain categories of genes, because fitness effects can only be detected if they are consistent over large evolutionary timescales [37]. To detect effects of single-site mutations on secondary metabolism, other genetic approaches than PICNC may be preferable: analysis of evolutionary divergence or balancing selection, association studies, and/or targeted mutagenesis [39, 40]. Despite this representation bias, predicted PNC will be useful to detect effects of deleterious alleles, segregating in natural populations (e.g., variants for carbon-metabolic genes in maize [41]) or fixed by genetic bottlenecks and/or transitions to selfing (e.g., during crop domestication) [37, 42]. Therefore, our approach could guide rapid cycling genomic selection and targeted mutagenesis for purging mutational load [43], especially in central carbon metabolism. Specifically, PICNC could target few candidate causal variants, for subsequent CRISPR-based base editing (e.g., C-to-T and A-to-G transitions) [44]. Compared to SIFT scores or other proxies for evolutionary constraint, predicted PNC would be especially useful in such applications, by targeting only a handful of sites to be edited simultaneously, for maximal effect on mutational load (Fig. 5) [45, 46].

Our approach was validated by cross-population genomic prediction (training in one set of populations, validation in a distinct set of populations). Compared to within-population genomic prediction, cross-population genomic prediction accuracies are typically much lower—and sometimes close to zero [47–53], because of differences in LD patterns and allele frequencies between training set and validation set [54–57]. Significant improvements in cross-population genomic prediction for GY suggest that prioritization of SNPs by predicted PNC could be useful for breeding applications (e.g., genomic pre-breeding [58, 59], or genomic selection in understudied populations [60–62]). They also suggest that predicted PNC could point to useful candidate causal variants, because accurate cross-population prediction requires very close tagging of causal variants by genomic markers [55]. Our improvements in prediction accuracy for GY (+5% and +38%) are on par with those achieved from genome-wide prioritization of genomic variants with many experimental annotations in large human samples (trans-ancestry predictions in cohorts of size > 150,000) [53]. However, they suggest that prioritization by PNC is only useful for fitness-related traits, for which causal variants are likely to be under evolutionary constraint. In this study, prioritization by PNC was tested in elite maize populations, in which deleterious mutations have been purged through sustained crop improvement [63]. It could be even more useful in other maize populations [64] or in other crop species, in which deleterious mutations are widespread, like sorghum [23, 65] or cassava [66].

Our approach exemplifies important benefits of this coming generation of protein structural machine learning annotations for predicting PNC without resorting to experimental data. These annotations may be produced by protein representation learning, using techniques like UniRep [13] or more recent models [67, 68]; they may also be produced by 3D structure prediction, using state-of-the-art models like AlphaFold 2 [69]. Our results will encourage future research, which will apply similar approaches to non-coding regulatory variants. Recent studies have introduced promising methods to predict gene regulation and infer high-resolution scores about the effect of mutations, e.g., for TF binding [70], RNA expression [71], and RNA-protein binding [72]. Such genomic annotations may be particularly useful to predict PNC in non-coding regions, because they can describe the impact of mutations on biological processes that are not directly related to the gene’s coding sequence. In contrast, the genomic annotations produced by UniRep described protein structural variation, using only protein sequence information [13]. As expected, these annotations were useful to describe fitness effects of nonsynonymous mutations, but they were not useful to predict the impact of mutations in UTRs, introns, or intergenic regions (Additional file 1: Fig. S5). Importantly, UniRep features actually decreased the accuracy of predicted PNC for mutations in these regions. Therefore, different sets of computational annotations should probably be selected to predict PNC at different categories of DNA sites.

Our results demonstrate the usefulness of our methodology. They also open possibilities for improved detection of fitness effects, by including broader sets of variants (e.g., non-coding variants), novel genomic annotations (e.g., regulatory effects of genes and mutations), and different evolutionary timescales (e.g., clade-specific fitness effects). Moreover, further improvements of SNP prioritization could be achieved by combining our approach with complementary techniques. Recent studies in human genetics have inferred relationships between genomic annotations and functional impact of mutations. These include methods based on evolutionary data, like CADD [8, 9] and LINSIGHT [11, 73], as well as methods based on summary statistics from genome-wide association studies (GWAS) [53, 74, 75]. GWAS-based methods are subject to biases from SNP survivorship and LD, but they describe the effect of mutations on explicitly defined traits. Therefore, these methods could be useful in combination with our proposed method, which does not suffer from the same caveats.

## Conclusions

To detect candidate causal variants at high resolution, we used nucleotide conservation and machine learning to predict the impact of mutations at single DNA sites. Our methodology benefited from genomic annotations which described protein structure by deep neural networks and estimated the structural impact of mutations by in silico mutagenesis. In maize, nucleotide conservation predicted by our approach performs better than observed nucleotide conservation. It results in significant functional enrichments and improves genomic prediction for grain yield across elite populations. Therefore, our approach (PICNC) could enable breeding applications which require the identification of candidate causal variants at high resolution, like cross-population genomic prediction and genome editing.

## Methods

### Training data

#### Genomic data

The B73 maize reference genome and its gene-model annotations under version 3 were downloaded from Ensembl Plants [76, 77]. Nuclear gene models with 3′UTR and 5′UTR annotations (hereafter, genes) were retained for further analyses (25,824 genes). The representative transcript for each gene model was the transcript with the most matches (bit-score > 50 in global alignment) with any other transcripts in the genomes of B73, Mo17, BTx623 (*Sorghum bicolor*), and Yugu1 (*Setaria italica*), or, by default, the longest transcript. Mutations in the coding region of representative transcripts were characterized at two types of DNA bases: monomorphic sites and SNP sites. Mutations at monomorphic sites were 20,136,310 random nonsynonymous substitutions in the maize genome at the selected genes, while those at SNP sites were the 483,448 observed nonsynonymous substitutions in a reference panel representative of inbred lines in maize [15, 78].

#### Evolutionary constraint

Publicly available data from a multiple-sequence alignment (MSA) across angiosperms was previously published in maize [14, 79]: neutral scores (depth of MSA at each site) and conservation scores (rejected substitutions) from gerp++ [4]. For each site *j*, phylogenetic nucleotide conservation (PNC) *w*_*j*_ was binary: *w*_*j*_ = 1 if the neutral score (tree size) was > 5 and the ratio of conservation score to neutral score was > 0.95 (i.e., substitution rate < 0.05), *w*_*j*_ = 0 otherwise.

#### Genomic annotations

Each mutation in coding regions was characterized by genomic structure (GC content, *k*-mer frequency and transposon insertion) and protein structure (mutation type, SIFT score, UniRep variables, and in silico mutagenesis scores).

GC content was the number of G or C bases from −49 to +50 bases from the site of the mutation. *k*-mer frequency was the average frequency of all 13-mers comprising the mutation’s site, calculated by jellyfish [80]. Predictions of transposon insertion at the mutation’s site (helitron, TIR, LINE, or LTR) were downloaded from GitHub [36, 81].

Mutation type (missense, STOP gain, or STOP loss), SIFT score, and SIFT class (“constrained” if SIFT score ≤ 0.05, “tolerated” otherwise) were predicted using SIFT 4G [3, 82, 83]. UniRep variables were the 256 values generated for each protein sequence by the “256-unit UniRep model” available from GitHub [13, 84]. In silico mutagenesis scores measured the impact of each mutation on proteins, as quantified by the UniRep variables: 256 deviations + 1 Euclidean distance between the reference representation and the mutated representation.

### PICNC: prediction of evolutionary constraint by genomic annotations

#### Model fitting

The relationship between genomic annotations and observed PNC (*w*_*j*_ = 0 or 1) was estimated by probability random forests [85, 86] implemented in the R package *ranger* [87]. To maximize power to differentiate negative (*w*_*j*_ = 0) and positive examples (*w*_*j*_ = 1) of evolutionary constraint, *w*_*j*_ was set to missing in intermediate cases where substitution rate > 0.05 or tree size < 5 (i.e., *w*_*j*_ = 0 only in least conserved regions where the MSA is missing). The probability *P*(*w*_*j*_ = 1) was estimated by 1000 trees per forest, 50,000 sites per tree (sampled with replacement), and at least 100 sites at each terminal node. Mutation effect, SIFT score, and SIFT class were always included as baseline predictors, while a third of remaining genomic annotations (GC content, *k*-mer frequency, transposon insertion, UniRep variables and in silico mutagenesis scores) were randomly sampled as predictors for each tree, based on recommendations for regression random forests [88]. To account for imbalance with respect to PNC and chromosome, each observation (site) was weighted by the inverse of the count of its respective class, as determined by its observed PNC and its chromosome.

#### Leave-one-chromosome-out prediction

For each chromosome *k* = 1, …, 10, PNC at each SNP site in chromosome *k* was predicted by a probability random forest (${\hat{w}}_j=\hat{P}\left({w}_j=1\right)$), trained on monomorphic sites in all chromosomes except *k* (Fig. 2). Importance of genomic annotations in random forests was estimated by the corrected impurity measure [18]. Classification accuracy was estimated by the percentage of sites for which ${\hat{w}}_j$(rounded) equaled *w*_*j*_, weighted by the sample weights (as described above). When estimating the importance of genomic annotations and assessing the effect of random forest parameters on classification accuracy (sets of genomic annotations used in prediction, proportions of genomic annotations sampled per tree, number of trees per forest), random forests were validated at monomorphic sites in chromosome 8 and trained (at monomorphic sites) in remaining chromosomes (Fig. 2).

In leave-one-chromosome-out prediction, alternate numbers of trees per forest (*n*_trees_=100, 250, 500, 1000) and alternate proportions of genomic annotations sampled per tree (*p*_variables_=$\frac{1}{12}$, $\frac{1}{6}$, $\frac{1}{3}$, $\frac{2}{3}$) were tested by hyperparameter tuning: for each left-out chromosome *k*, optimal hyperparameters *n*_trees_ and *p*_variables_ were chosen to maximize weighted classification accuracy, by training in half of the chromosomes (all odd chromosomes when *k* was even, and vice versa) and validation in the remaining chromosomes (excluding *k*). For each left-out chromosome *k*, the optimal hyperparameters were *n*_trees_=1000 (as many trees as allowed) and *p*_variables_=$\frac{1}{3}$ (recommended value in regression random forest [88]).

#### Validation in sorghum genome

Validation in the sorghum genome consisted of fitting the PICNC model in the whole maize genome, as described above (see “Model fitting”), and predicting PNC at sorghum SNPs based on genomic annotations in the BTx623 sorghum reference genome.

Sorghum SNPs were the polymorphisms previously identified in a diverse panel of 499 lines, described by Lozano et al. [23, 89]. The BTx623 sorghum reference genome and its gene-model annotations were downloaded from Phytozome under version 3.1, release 313 [90, 91]. Genomic annotations in sorghum were the same as in maize, but did not include annotations about transposon insertion (helitron, TIR, LINE, LTR), because these were not available in sorghum and not important in maize (Fig. 3C). Mutation type, SIFT score and SIFT class were predicted using the SIFT database from Lozano et al. [23, 89]. GC content, *k*-mer frequency, Unirep variables, and in silico mutagenesis scores were computed in version 3.1 of the sorghum reference genome, as described above (“Genomic annotations”).

### Validation of predicted evolutionary constraint

#### Experimental SNP annotations

Predicted PNC in maize was validated by measures of functional importance of SNPs: within-species conservation, *cis* eQTL effect, and chromatin accessibility. Within-species conservation was quantified by minor allele frequency (MAF), estimated in a filtered set of SNPs (bi-allelic, minor allele count ≥ 3, missingness ≤ 50%) in the Hapmap 3.2.1 reference panel [15], imputed by BEAGLE 5.0 [92]. *Cis* eQTL effects were the statistical associations (in absolute value) between SNPs and 3′ RNA-seq expression of genes, in a diverse panel of 299 inbred lines [22, 93]. *Cis* eQTL effects in germinating shoot or germinating root were estimated for the SNPs with minor allele frequency ≥ 0.05 in this panel, in a linear regression model including the PEER factors from [22] as covariates, using GEMMA 0.98.1 [94]. Chromatin accessibility was characterized by hotspots of MNase hypersensitivity in germinating shoot or germinating root [21, 95].

In maize, PNC was validated by experimental SNP annotations in a generalized additive model fitted in the R package *mgcv* [96]. PNC was regressed on MAF and *cis* eQTL effects (by cubic regression splines), and chromatin accessibility (as factors), while accounting for chromosome (as factor) and whether the site was included in the MSA (as factor, to control for bias of the MSA towards gene-dense regions).

In sorghum, predicted PNC was validated by MAF, calculated in the diverse panel of Lozano et al. [23, 89].

#### Experimental gene annotations

Predicted PNC in maize was validated by gene properties: gene expression, gene ontology, pathway annotation, and number of segregating SNPs. Gene expression was quantified by RNA abundance across 23 tissues, and protein abundance across 32 tissues [29]. In all analyses, gene expression was log-transformed: *log*(*x* + 1) where *x* is RNA abundance in fragments per kilobase of transcript per million mapped reads (FPKM) or protein abundance in distributed normalized spectral abundance factor (dNSAF). Experimentally validated gene ontology (GO) annotations [97] were retrieved by mapping protein sequences to the eggNOG database, using DIAMOND [98]. In enrichment analyses, GO annotations were trimmed to the broader (and less redundant) GO slim terms in the “plant GO slim” subset [30], and GO annotations with fewer than 20 positives were discarded (87 selected GO terms). Pathway annotations were retrieved from CornCyc, release 2021/03/25 [31] (Additional file 2: Table S1). The numbers of segregating nonsynonymous SNPs (*P*_n_) and segregating synonymous SNPs (*P*_s_) were based on a maize reference panel (MAF ≥ 0.01 in Hapmap 3.2.1). The ratio *P*_n_/*P*_s_ and proportion *P*_n_/(*P*_n_+*P*_s_) were calculated for each gene with enough observed segregating synonymous SNPs (*P*_s_ ≥ 5).

In sorghum, predicted PNC was validated by the number of segregating SNPs: ratio *P*_n_/*P*_s_ and proportion *P*_n_/(*P*_n_+*P*_s_) calculated in the diverse panel of Lozano et al. [23, 89].

In validations by experimental gene annotations, genes containing sites with ${\hat{w}}_j$ above a threshold value were selected. Threshold values were the 50%, 90%, 99%, and 99.9% percentiles of ${\hat{w}}_j$’s. Using these successive selections, we assessed the functional enrichment of prioritized genes as fewer sites were included due to more stringent thresholds. The significance of the enrichment for gene expression (difference in mean expression between selected genes and all genes) and GO slim terms (overrepresentation of term among selected genes) were tested by two-sample *t*-test and Fisher’s exact test, respectively.

#### Field traits in hybrid maize

Two panels of hybrid maize lines were analyzed to assess the usefulness of predicted PNC for genomic prediction: a diversity panel (Ames-H; *n*=1106) and a collection of biparental crosses having B73 as their common parent (NAM-H; *n*=1640) [16, 99]. These panels were phenotyped for three agronomic traits: days to silking (DTS), plant height (PH), and grain yield adjusted for DTS (GY). They were genotyped for 12,659,487 genome-wide SNPs, including *m*=103,905 nonsynonymous SNPs in the coding regions of the 25,824 genes selected in this study.

Predicted PNC (${\hat{w}}_j$) was used to weight each nonsynonymous SNP *j* in genomic prediction models, fitted in hybrid maize panels:

$${\displaystyle \begin{array}{l}\mathbf{y}=\mathbf{Q}\boldsymbol{\alpha } +\mathbf{u}+{\mathbf{u}}_{CDS}+\mathbf{e}\\ {}\mathbf{u}\sim N\left(\mathbf{0},\mathbf{G}{\sigma}_u^2\right)\\ {}\begin{array}{l}{\mathbf{u}}_{CDS}\sim N\left(\mathbf{0},{\mathbf{G}}_{CDS}{\sigma}_{CDS}^2\right)\\ {}\mathbf{e}\sim N\left(\mathbf{0},\mathbf{I}{\sigma}_e^2\right),\end{array}\end{array}}$$

where **y** is the *n*-vector of mean phenotypic values; **Q** is a *n* × 4 matrix depicting population structure by a column of ones (for the intercept) and the three principal components from the Hapmap 3.2.1 panel, with respective effects **α**; **e** is the vector of errors; **G** is the *n* × *n* genome-wide relationship matrix such that the *n*-vector **u** consists of genome-wide breeding values:

$${g}_{i{i}^{\prime }}=\frac{\sum_l\left({x}_{il}-2{p}_l\right)\left({x}_{i^{\prime }l}-2{p}_l\right)}{\sum_l2{p}_l\left(1-{p}_l\right)},$$

where *x*_*il*_ is the genotype of hybrid *i* at genome-wide SNP *l*, *p*_*l*_ is the estimated frequency of SNP *l* in hybrid panels.

**G**_CDS_ is the *n* × *n* relationship matrix from nonsynonymous SNPs weighted by predicted PNC, such that the *n*-vector **u**_*CDS*_ consists of breeding values due to weighted nonsynonymous SNPs:

$${\displaystyle \begin{array}{l}{\mathbf{G}}_{CDS}=\frac{{\mathbf{X}}_{CDS}\mathbf{W}{\mathbf{X}}_{CDS}^{\mathrm{T}}}{\sum_{j=1}^m{\hat{w}}_j}\\ {}\mathbf{W}=\mathit{\operatorname{diag}}{\left\{{\hat{w}}_j\right\}}_{j=1,\dots, m},\end{array}}$$

where **X**_CDS_ is the *n* × *m* matrix of genotypes at nonsynonymous SNPs.

Genomic prediction models were fitted by REML, using the R package *qgg* [100]. Genomic prediction accuracy was estimated by the Pearson correlation between predicted and observed phenotypic values:

$$cor\left(\hat{\mathbf{y}},\mathbf{y}\right);\hat{\mathbf{y}}=\mathbf{Q}\hat{\boldsymbol{\alpha}}+\hat{\mathbf{u}}+{\hat{\mathbf{u}}}_{CDS}$$

In validations of predicted PNC by genomic prediction, ${\hat{w}}_j$’s below a threshold value were set to zero. Threshold values were the 0%, 50%, 90%, 99%, and 99.9% percentiles of ${\hat{w}}_j$’s, among the *m* SNPs observed in hybrid panels. Using these successive truncations, we assessed the enrichment of prioritized SNPs for genomic effects, as fewer of them were included due to more stringent thresholding on their weights.

The significance of ${\hat{w}}_j$’s as useful weights in genomic prediction was tested by comparing genomic prediction accuracy with the accuracies achieved by 20 random permutations of ${\hat{w}}_j$’s, hence testing the null hypothesis that ${\hat{w}}_j$’s are as useful as expected by chance. For each permutation *b*, ${\hat{w}}_j$’s were randomly shuffled, and the vector of permuted weights ${\hat{w}}_j^{(b)}$ was used to weight and prioritize SNPs, then calculate genomic prediction accuracy $cor\left({\hat{\mathbf{y}}}^{(b)},\mathbf{y}\right)$ as described above. The improvement of genomic prediction accuracy from actual weights ${\hat{w}}_j$’s was deemed significant (*P* < 0.05) if $cor\left(\hat{\mathbf{y}},\mathbf{y}\right)> cor\left({\hat{\mathbf{y}}}^{(b)},\mathbf{y}\right)$ for all *b* = 1, …, 20.

## Supplementary Information

Additional file 1: **Figure S1.** Distribution of neutral tree size (expected number of nucleotide substitutions under a neutral model) by category of DNA bases. **Figure S2.** Classification accuracy for different hyperparameters of the probability random forest in PICNC. **Figure S3.** Prediction accuracy of regression random forests for experimental gene annotations, by UniRep variables. **Figure S4.** Concordance between importance of protein features (UniRep variables) for phylogenetic nucleotide conservation (PNC) across species and measures of gene variability within species. **Figure S5.** Classification accuracy for PNC for different categories of DNA sites: CDS (nonsynonymous mutations), Intron+UTR, Intergenic. **Figure S6.** Difference in SNP variability at genes prioritized by SIFT score or phylogenetic nucleotide conservation (PNC), in maize or sorghum. **Figure S7.** Enrichment of genes prioritized by SIFT conservation (1 − SIFT score) or phylogenetic nucleotide conservation (PNC), for gene ontology (GO) classes. **Figure S8.** Prioritization of nonsynonymous SNPs in genomic prediction for agronomic traits in hybrid panels.

Additional file 2: **Table S1.** Functional information about gene models in the maize reference genome: experimental gene annotations and percentile for SIFT conservation and phylogenetic nucleotide conservation (PNC). **Table S2.** Number of genes prioritized by SIFT conservation or phylogenetic nucleotide conservation (PNC), in each CornCyc pathway. **Table S3.** Prioritization of nonsynonymous SNPs by SIFT conservation or phylogenetic nucleotide conservation (PNC): number of selected SNPs in hybrid panels and expected number of putative deleterious mutations per inbred line based on minor allele frequency (MAF) in reference panel.

Additional file 3. Review history.

## Data Availability

The scripts used to generate the results are publicly available in BitBucket under an MIT license [101], and in Zenodo under a Creative Commons Attribution 4.0 International license [102]. The datasets and models generated in the current study are publicly available in CyVerse under Public Domain Dedication and License v1.0 [103]. Third-party data sets used in this study include the following: reference genome assembly and annotations, in maize B73 (version 3) from Ensembl Plants [76, 77], and in sorghum BTx623 (version 3.1, release 313) from Phytozome [90, 91]; genomic data in reference maize panel, from CyVerse [78]; genomic and transcriptomic data in diverse maize inbred lines, from CyVerse [93]; genomic and phenotypic data in diverse hybrid maize lines, from CyVerse [99]; neutral scores and conservation scores from gerp++, from Dryad [79]; transposon insertion annotations in maize B73, from GitHub [81]; MNase-seq data in maize B73 seedlings, from NCBI SRA [95]; Plant GO slim subset [30].
