# Supplementary material for: Prediction of evolutionary constraint by genomic annotations improves functional prioritization of genomic variants in maize
Source: Genome Biol. 2022 Sep 1;23:183. doi: 10.1186/s13059-022-02747-2 (PMC9438327; doi:10.1186/s13059-022-02747-2)
Supplement: Supplementary file 1 — Additional file 1: Figure S1. Distribution of neutral tree size (expected number of nucleotide substitutions under a neutral model) by category of DNA bases. Figure S2. Classification accuracy for different hyperparameters of the probability random forest in PICNC. Figure S3. Prediction accuracy of regression random forests for experimental gene annotations, by UniRep variables. Figure S4. Concordance between importance of protein features (UniRep variables) for phylogenetic nucleotide conservation (PNC) across species and measures of gene variability within species. Figure S5. Classification accuracy for PNC for different categories of DNA sites: CDS (nonsynonymous mutations), Intron+UTR, Intergenic. Figure S6. Difference in SNP variability at genes prioritized by SIFT score or phylogenetic nucleotide conservation (PNC), in maize or sorghum. Figure S7. Enrichment of genes prioritized by SIFT conservation (1 − SIFT score) or phylogenetic nucleotide conservation (PNC), for gene ontology (GO) classes. Figure S8. Prioritization of nonsynonymous SNPs in genomic prediction for agronomic traits in hybrid panels. [file 13059_2022_2747_MOESM1_ESM.pdf]

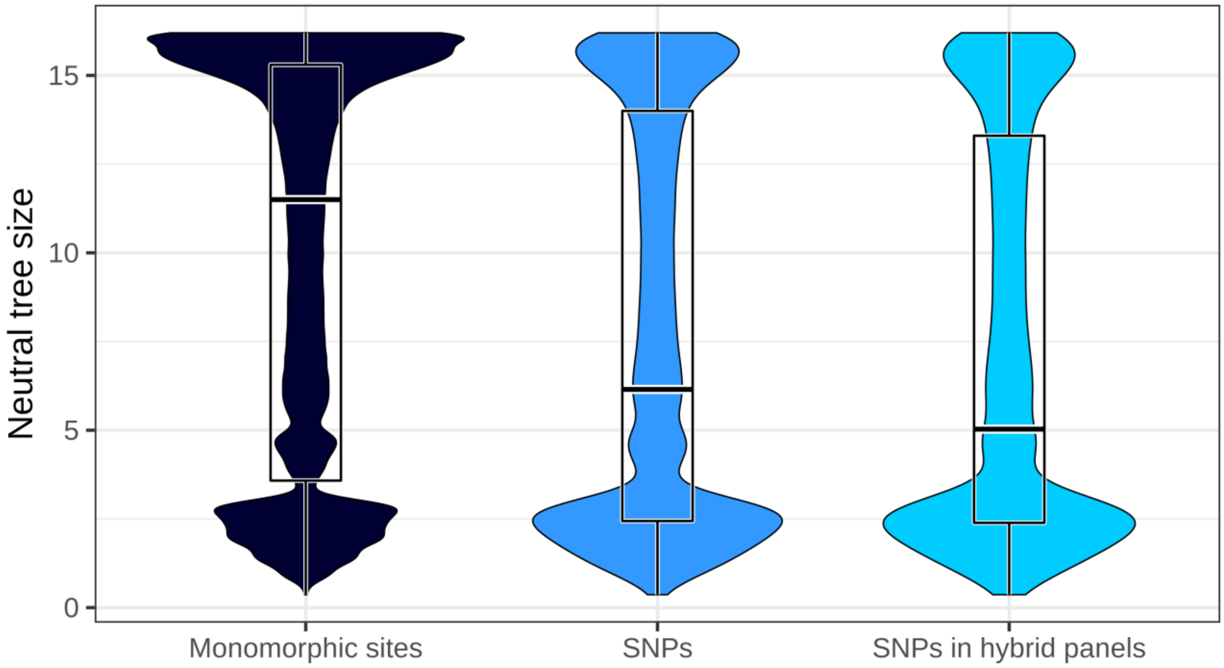

Fig. S1 Distribution of neutral tree size (expected number of nucleotide substitutions under a neutral model) by category of DNA bases. Neutral tree size was calculated as previously described [14]. Monomorphic sites: sites with no observed polymorphism within maize. SNPs: observed polymorphisms in Hapmap 3.2.1, a representative panel of inbred lines in maize [15]. SNPs in hybrid panels: subset of SNPs observed in Hapmap 3.2.1, which are also observed in two panels of hybrid crosses between inbred lines and testers [16].

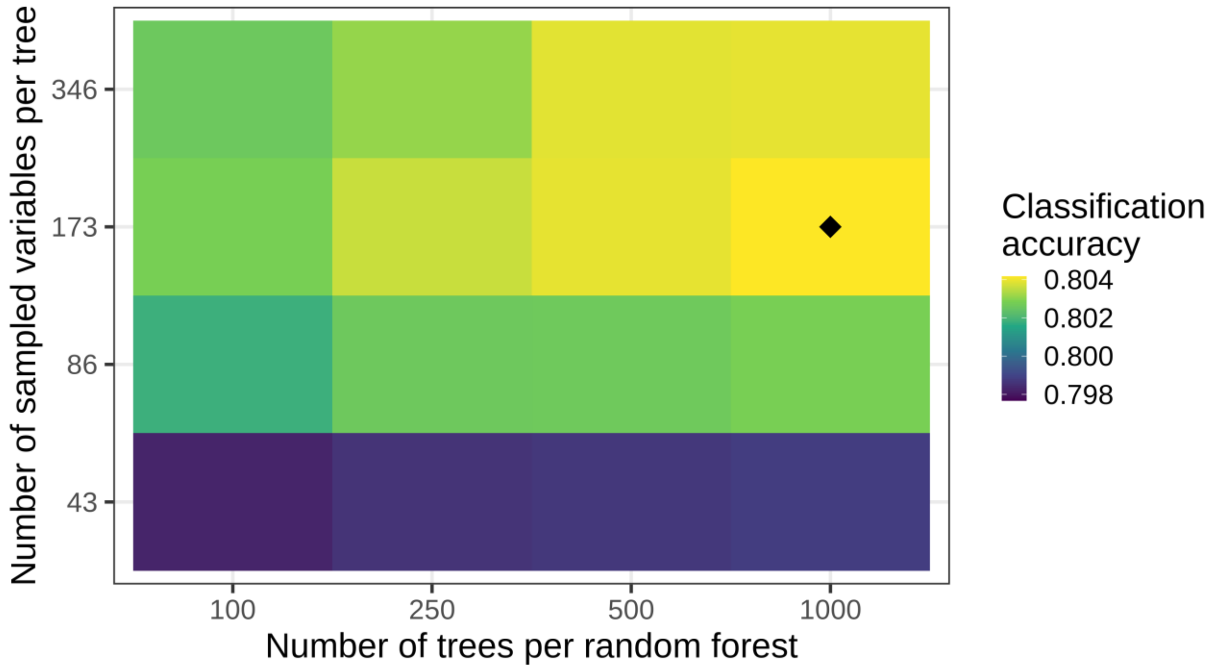

Fig. S2 Classification accuracy for different hyperparameters of the probability random forest in PICNC. Accuracy: percentage of correct calls, i.e., the percentage of sites in chromosome 8 for which predicted PNC (rounded) equaled observed PNC, over three replicates. Accuracy was weighted to account for imbalance with respect to PNC (see Methods). Hyperparameters in the probability random forest were the number of trees in the probability random forest (*x*-axis) and the number of sampled variables per tree (*y*-axis).

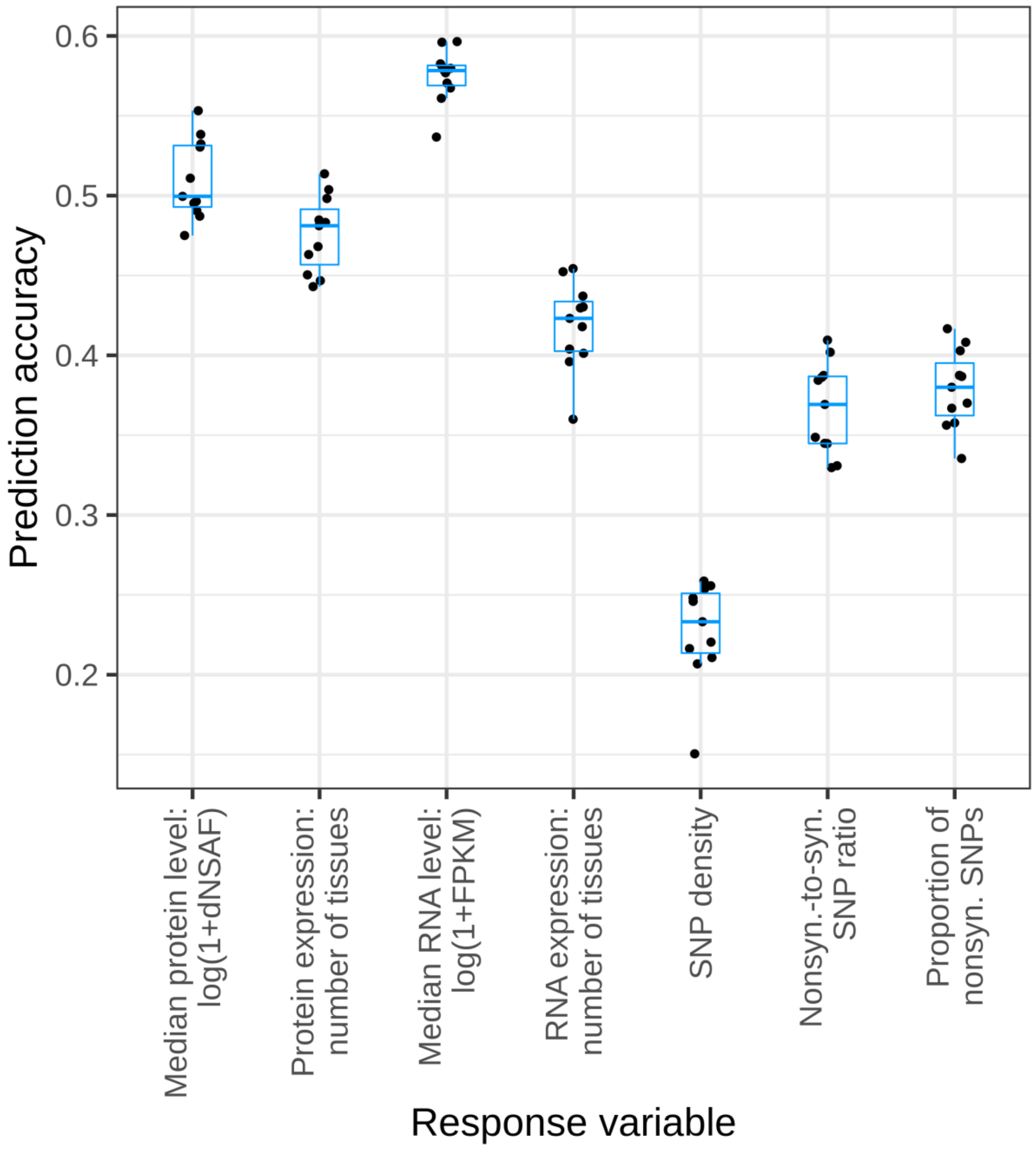

Fig. S3 Prediction accuracy of regression random forests for experimental gene annotations, by UniRep variables. UniRep variables are generated by the 256-unit UniRep model. Prediction accuracy is the Pearson correlation coefficient between predicted values and observed values. Expression is quantified by RNA abundance (over 23 tissues) and protein abundance (over 32 tissues) based on the gene expression atlas of [94]: median expression, and number of tissues with non-zero expression level. SNP density: percentage of segregating SNP sites in genes (MAF $\geq$ 0.01 in Hapmap 3.2.1). Nonsyn.-to-syn. SNP ratio: ratio of nonsynonymous-to-synonymous SNPs (P_n/P_s) within each gene. Proportion of nonsyn. SNPs: fraction of nonsynonymous SNPs over the total number of SNPs ($P_n/(P_n+P_s)$) within each gene.

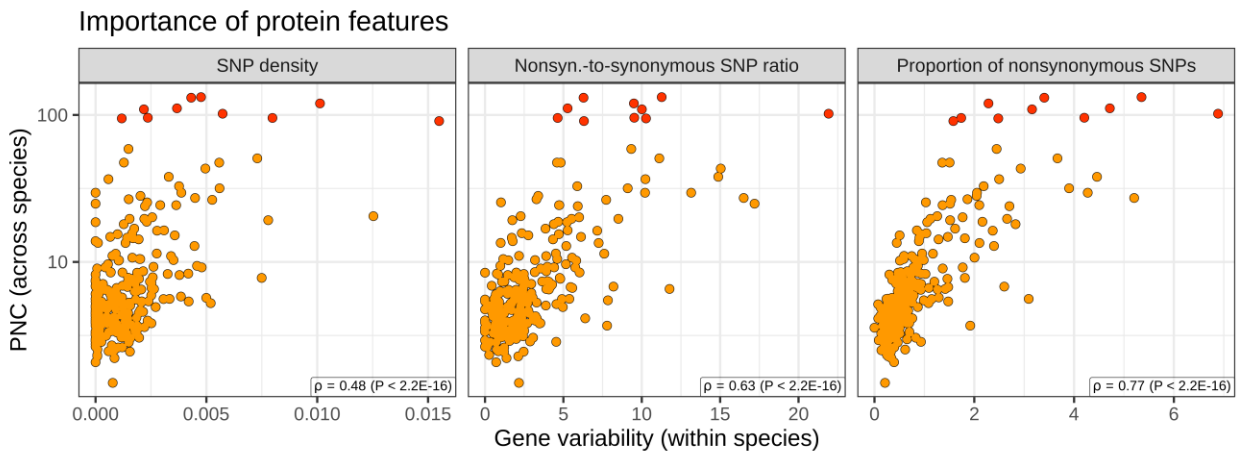

Fig. S4 Concordance between importance of protein features (UniRep variables) for phylogenetic nucleotide conservation (PNC) across species and measures of gene variability within species. Importance of protein features was inferred from the full PICNC model (*y*-axis) and from random forest models regressing measures of gene variability on protein features (*x*-axis). SNP density: percentage of segregating SNP sites in genes (MAF ≥ 0.01 in Hapmap 3.2.1). Nonsyn.-to-synonymous SNP ratio: ratio of nonsynonymous-to-synonymous SNPs (P_n/P_s) within each gene. Proportion of nonsynonymous SNPs: fraction of nonsynonymous SNPs over the total number of SNPs ($P_n/(P_n+P_s)$) within each gene. ρ: Spearman correlation coefficient.

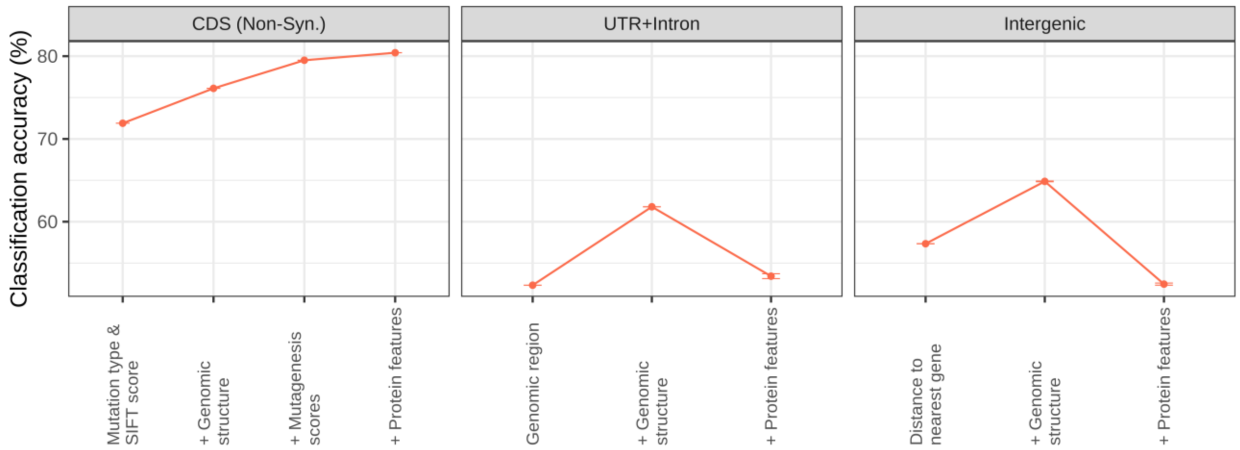

Fig. S5 Classification accuracy for PNC for different categories of DNA sites. Accuracy: percentage of correct calls, i.e., the percentage of sites in chromosome 8 for which predicted PNC (rounded) equaled observed PNC, over three replicates. Accuracy was weighted to account for imbalance with respect to PNC (see Methods). CDS (Non-Syn.): nonsynonymous mutations in protein coding regions. UTR+Intron: mutations in non-coding parts of the transcribed region: 3' untranslated region (UTR), 5' UTR, and introns. Intergenic: mutations within 1 kb of transcribed regions. Mutation type & SIFT score: Mutation type (missense, STOP gain or STOP loss), SIFT score (with missing values set to 1) and SIFT class ("constrained" if SIFT score ≤ 0.05, "tolerated" otherwise). Genomic structure: GC content, k-mer frequency, transposon insertion. Mutagenesis scores: *in silico* mutagenesis scores for UniRep variables. Protein features: UniRep variables, generated by the 256-unit UniRep model; intergenic mutations were described by the protein features of the nearest gene. Distance to the nearest gene: distance between the mutation and the boundary of the nearest gene.

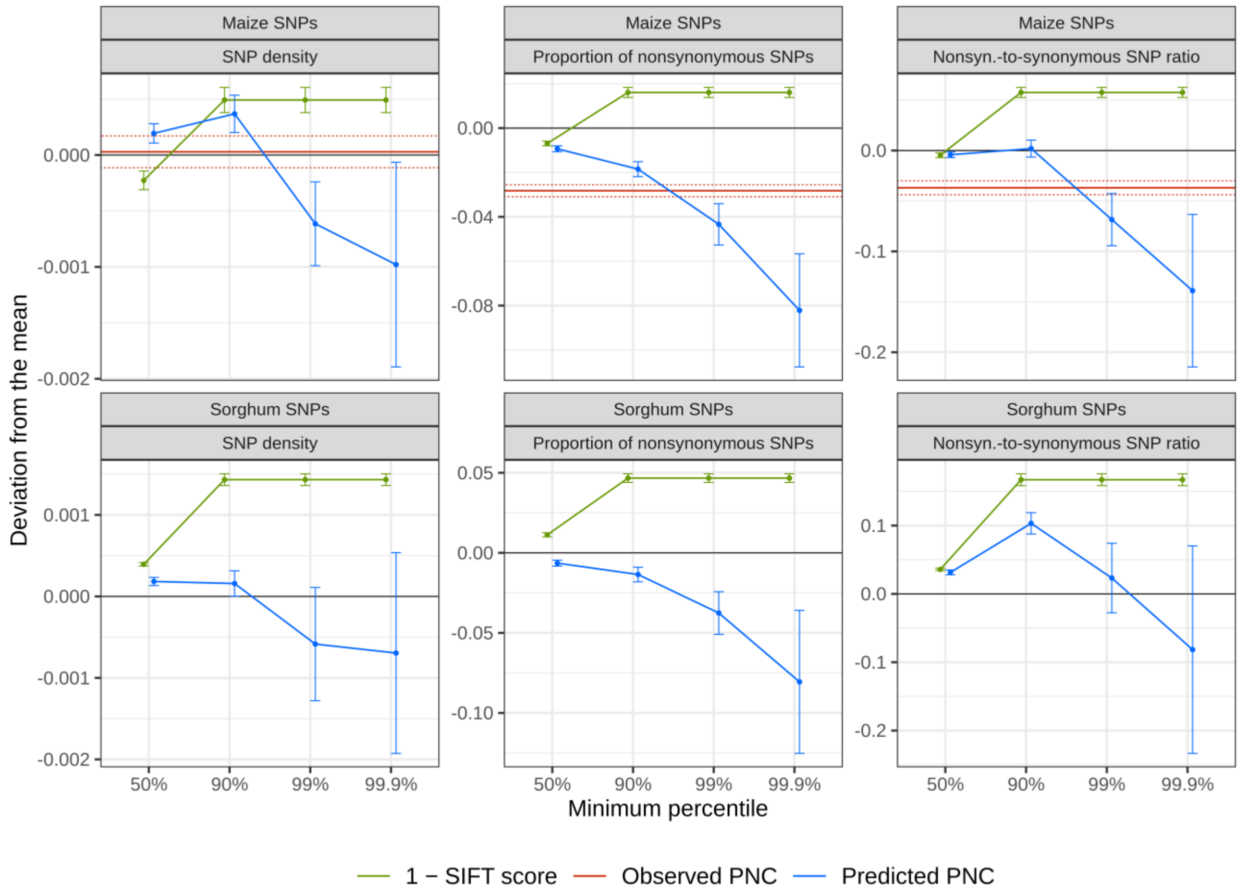

Fig. S6 Difference in SNP variability at genes prioritized by SIFT score or phylogenetic nucleotide conservation (PNC), in maize or sorghum. Maize SNPs: observed polymorphisms in the Hapmap 3.2.1 panel [15]; Sorghum SNPs: observed polymorphisms in the sorghum haplotype panel of Lozano *et al.* (2021) [21]. Difference in gene annotations between prioritized genes and all genes (*y*-axis); SNP density: percentage of segregating SNP sites in genes (MAF ≥ 0.01 in Hapmap 3.2.1); Proportion of nonsynonymous SNPs: fraction of nonsynonymous SNPs over the total number of SNPs ($P_n/(P_n+P_s)$) within each gene; Nonsyn.-to-synonymous SNP ratio: ratio of nonsynonymous-to-synonymous SNPs (P_n/P_s) within each gene. Genes were prioritized by selecting SNPs with SIFT conservation (1 – SIFT score) or predicted PNC above the 50%, 90%, 99%, or 99.9% percentile, or observed PNC equal to 1 (tree size > 5, substitution rate < 0.05). Error bars and dotted lines represent 95% confidence intervals in two-sample t-tests, for SIFT score or predicted PNC, and observed PNC, respectively.

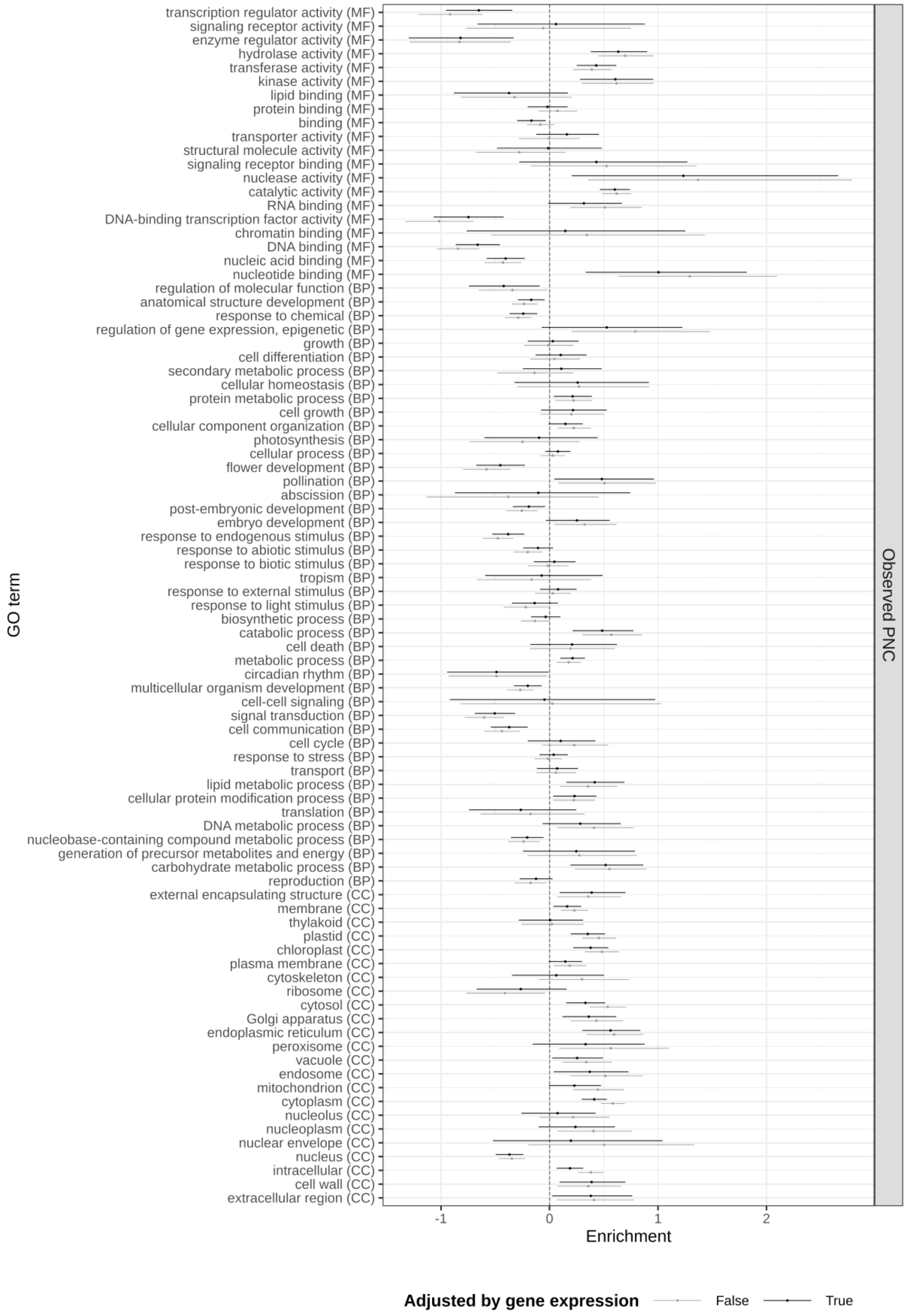

transcription regulator activity (MF)
signaling receptor activity (MF)
enzyme regulator activity (MF)
hydrolase activity (MF)
transferase activity (MF)
kinase activity (MF)
lipid binding (MF)
protein binding (MF)
binding (MF)
transporter activity (MF)
structural molecule activity (MF)
signaling receptor binding (MF)
nuclease activity (MF)
catalytic activity (MF)
RNA binding (MF)
DNA-binding transcription factor activity (MF)
chromatin binding (MF)
DNA binding (MF)
nucleic acid binding (MF)
nucleotide binding (MF)
regulation of molecular function (BP)
anatomical structure development (BP)
response to chemical (BP)
regulation of gene expression, epigenetic (BP)
growth (BP)
cell differentiation (BP)
secondary metabolic process (BP)
cellular homeostasis (BP)
protein metabolic process (BP)
cell growth (BP)
cellular component organization (BP)
photosynthesis (BP)
cellular process (BP)
flower development (BP)
pollination (BP)
abscission (BP)
post-embryonic development (BP)
embryo development (BP)
response to endogenous stimulus (BP)
response to abiotic stimulus (BP)
response to biotic stimulus (BP)
tropism (BP)
response to external stimulus (BP)
response to light stimulus (BP)
biosynthetic process (BP)
catabolic process (BP)
cell death (BP)
metabolic process (BP)
circadian rhythm (BP)
multicellular organism development (BP)
cell-cell signaling (BP)
signal transduction (BP)
cell communication (BP)
cell cycle (BP)
response to stress (BP)
transport (BP)
lipid metabolic process (BP)
cellular protein modification process (BP)
translation (BP)
DNA metabolic process (BP)
nucleobase-containing compound metabolic process (BP)
generation of precursor metabolites and energy (BP)
carbohydrate metabolic process (BP)
reproduction (BP)
external encapsulating structure (CC)
membrane (CC)
thylakoid (CC)
plastid (CC)
chloroplast (CC)
plasma membrane (CC)
cytoskeleton (CC)
ribosome (CC)
cytosol (CC)
Golgi apparatus (CC)
endoplasmic reticulum (CC)
peroxisome (CC)
vacuole (CC)
endosome (CC)
mitochondrion (CC)
cytoplasm (CC)
nucleolus (CC)
nucleoplasm (CC)
nuclear envelope (CC)
nucleus (CC)
intracellular (CC)
cell wall (CC)
extracellular region (CC)
GO term
-1
0
1
2
Enrichment
Observed PNC
Adjusted by gene expression
False
True

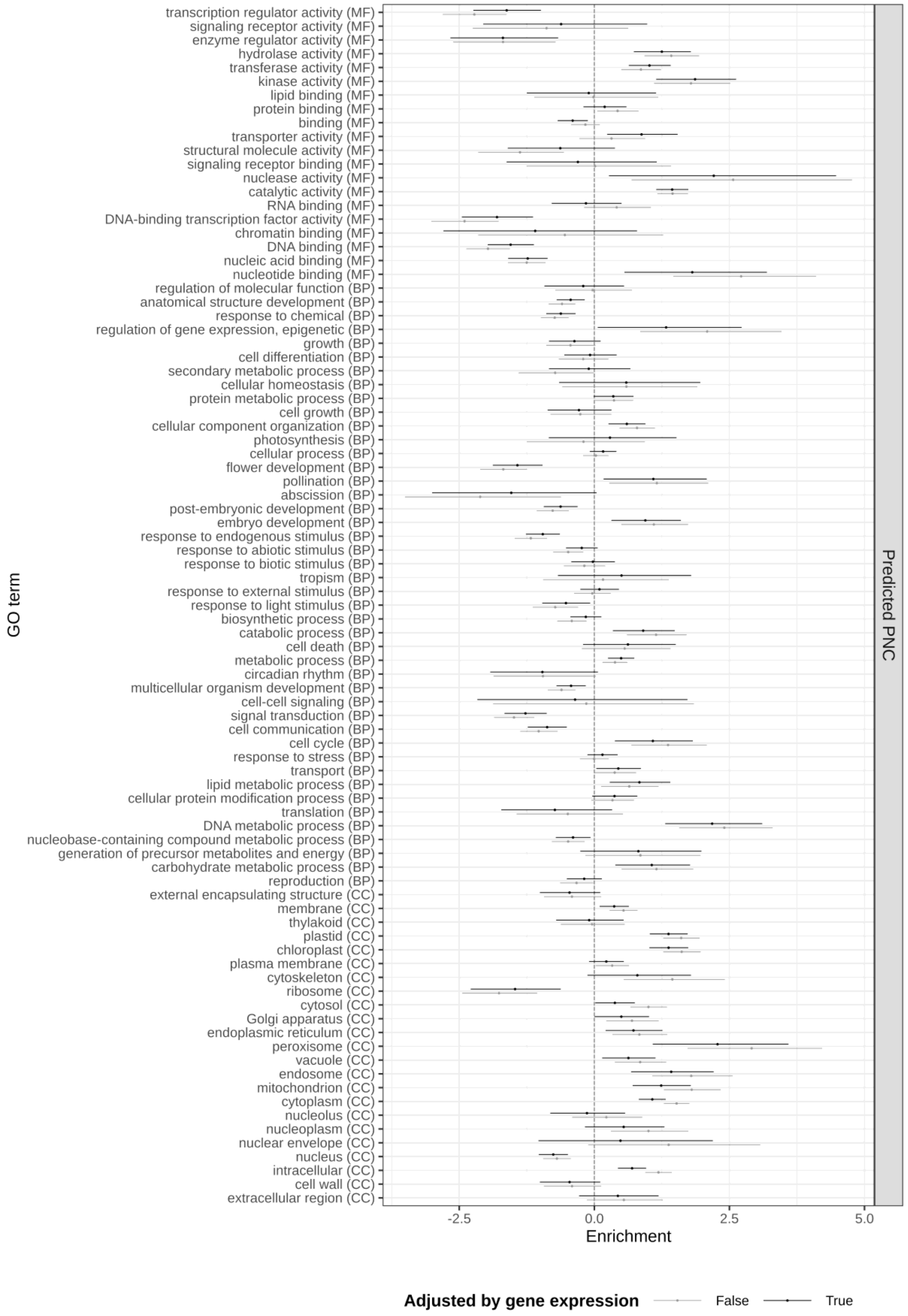

GO term
transcription regulator activity (MF)
signaling receptor activity (MF)
enzyme regulator activity (MF)
hydrolase activity (MF)
transferase activity (MF)
kinase activity (MF)
lipid binding (MF)
protein binding (MF)
binding (MF)
transporter activity (MF)
structural molecule activity (MF)
signaling receptor binding (MF)
nuclease activity (MF)
catalytic activity (MF)
RNA binding (MF)
DNA-binding transcription factor activity (MF)
chromatin binding (MF)
DNA binding (MF)
nucleic acid binding (MF)
nucleotide binding (MF)
regulation of molecular function (BP)
anatomical structure development (BP)
response to chemical (BP)
regulation of gene expression, epigenetic (BP)
growth (BP)
cell differentiation (BP)
secondary metabolic process (BP)
cellular homeostasis (BP)
protein metabolic process (BP)
cell growth (BP)
cellular component organization (BP)
photosynthesis (BP)
cellular process (BP)
flower development (BP)
pollination (BP)
abscission (BP)
post-embryonic development (BP)
embryo development (BP)
response to endogenous stimulus (BP)
response to abiotic stimulus (BP)
response to biotic stimulus (BP)
tropism (BP)
response to external stimulus (BP)
response to light stimulus (BP)
biosynthetic process (BP)
catabolic process (BP)
cell death (BP)
metabolic process (BP)
circadian rhythm (BP)
multicellular organism development (BP)
cell-cell signaling (BP)
signal transduction (BP)
cell communication (BP)
cell cycle (BP)
response to stress (BP)
transport (BP)
lipid metabolic process (BP)
cellular protein modification process (BP)
translation (BP)
DNA metabolic process (BP)
nucleobase-containing compound metabolic process (BP)
generation of precursor metabolites and energy (BP)
carbohydrate metabolic process (BP)
reproduction (BP)
external encapsulating structure (CC)
membrane (CC)
thylakoid (CC)
plastid (CC)
chloroplast (CC)
plasma membrane (CC)
cytoskeleton (CC)
ribosome (CC)
cytosol (CC)
Golgi apparatus (CC)
endoplasmic reticulum (CC)
peroxisome (CC)
vacuole (CC)
endosome (CC)
mitochondrion (CC)
cytoplasm (CC)
nucleolus (CC)
nucleoplasm (CC)
nuclear envelope (CC)
nucleus (CC)
intracellular (CC)
cell wall (CC)
extracellular region (CC)
Predicted PNC
-2.5
0.0
2.5
5.0
Enrichment
Adjusted by gene expression
False
True

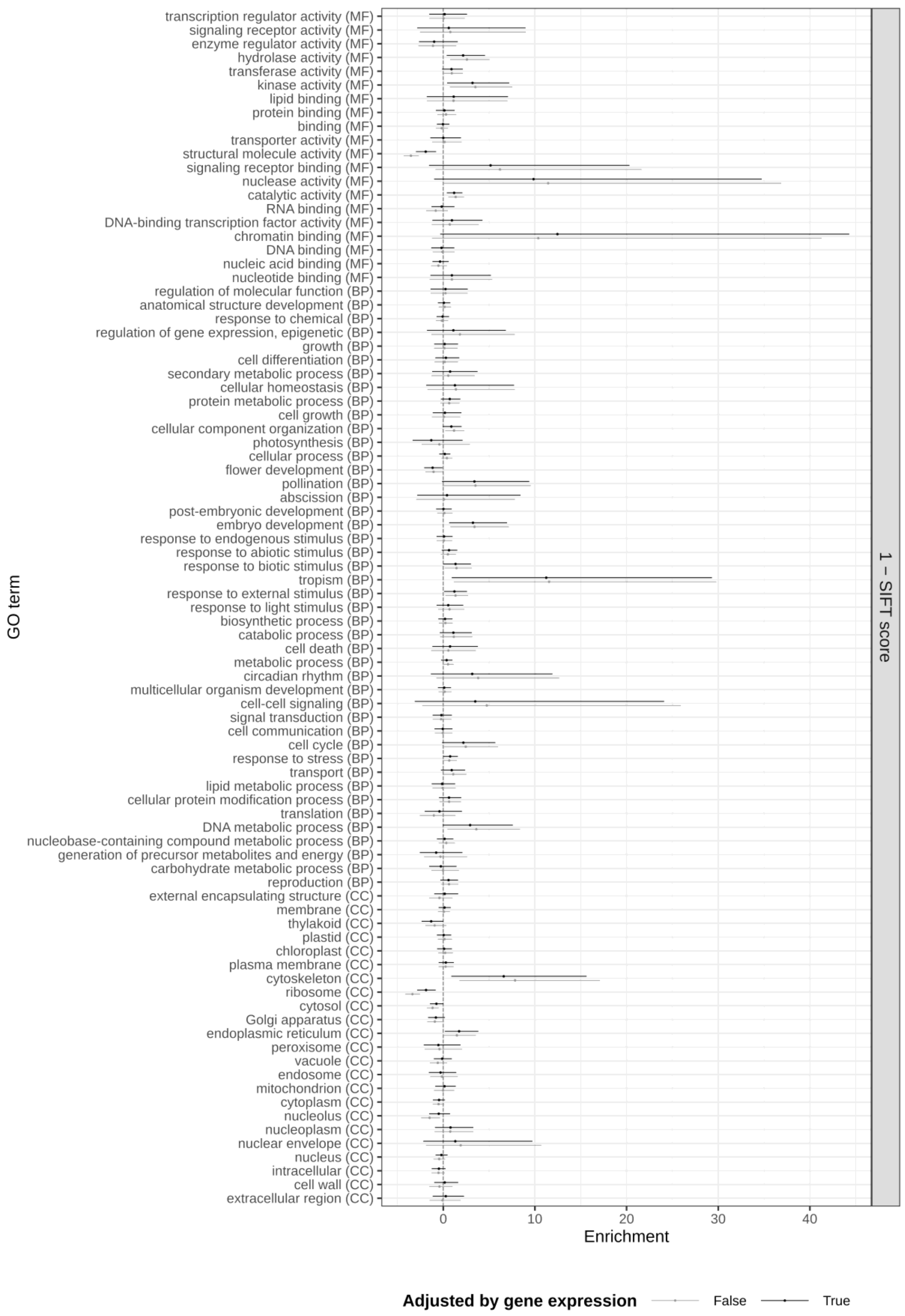
transcription regulator activity (MF)
signaling receptor activity (MF)
enzyme regulator activity (MF)
hydrolase activity (MF)
transferase activity (MF)
kinase activity (MF)
lipid binding (MF)
protein binding (MF)
binding (MF)
transporter activity (MF)
structural molecule activity (MF)
signaling receptor binding (MF)
nuclease activity (MF)
catalytic activity (MF)
RNA binding (MF)
DNA-binding transcription factor activity (MF)
chromatin binding (MF)
DNA binding (MF)
nucleic acid binding (MF)
nucleotide binding (MF)
regulation of molecular function (BP)
anatomical structure development (BP)
response to chemical (BP)
regulation of gene expression, epigenetic (BP)
growth (BP)
cell differentiation (BP)
secondary metabolic process (BP)
cellular homeostasis (BP)
protein metabolic process (BP)
cell growth (BP)
cellular component organization (BP)
photosynthesis (BP)
cellular process (BP)
flower development (BP)
pollination (BP)
abscission (BP)
post-embryonic development (BP)
embryo development (BP)
response to endogenous stimulus (BP)
response to abiotic stimulus (BP)
response to biotic stimulus (BP)
tropism (BP)
response to external stimulus (BP)
response to light stimulus (BP)
biosynthetic process (BP)
catabolic process (BP)
cell death (BP)
metabolic process (BP)
circadian rhythm (BP)
multicellular organism development (BP)
cell-cell signaling (BP)
signal transduction (BP)
cell communication (BP)
cell cycle (BP)
response to stress (BP)
transport (BP)
lipid metabolic process (BP)
cellular protein modification process (BP)
translation (BP)
DNA metabolic process (BP)
nucleobase-containing compound metabolic process (BP)
generation of precursor metabolites and energy (BP)
carbohydrate metabolic process (BP)
reproduction (BP)
external encapsulating structure (CC)
membrane (CC)
thylakoid (CC)
plastid (CC)
chloroplast (CC)
plasma membrane (CC)
cytoskeleton (CC)
ribosome (CC)
cytosol (CC)
Golgi apparatus (CC)
endoplasmic reticulum (CC)
peroxisome (CC)
vacuole (CC)
endosome (CC)
mitochondrion (CC)
cytoplasm (CC)
nucleolus (CC)
nucleoplasm (CC)
nuclear envelope (CC)
nucleus (CC)
intracellular (CC)
cell wall (CC)
extracellular region (CC)
GO term
0
10
20
30
40
Enrichment
1 – SIFT score
Adjusted by gene expression
False
True

Fig. S7 Enrichment of genes prioritized by SIFT conservation (1 – SIFT score) or phylogenetic nucleotide conservation (PNC), for gene ontology (GO) classes. Enrichment: effect of maximum conservation score (observed PNC, predicted PNC or SIFT conservation) in each protein coding sequence on the odds ratio for GO annotations [Pr(GO annotation)/(1 – Pr(GO annotation)], based on logistic regression. Estimated enrichments are shown on a log scale: point estimates (dot) and 95%-confidence intervals (segment). Gray symbols: effects of gene prioritizations are not adjusted (simple logistic regression of GO annotation on maximum PNC or SIFT conservation). Black symbols: effects of gene prioritizations are adjusted by gene expression (logistic regression including gene expression variables as covariates). Gene expression variables are RNA abundance (FPKM over 23 tissues) and protein abundance (dNSAF over 32 tissues) based on the gene expression atlas of [94]: median expression [median of log(1+FPKM) or log(1+dNSAF)], and number of tissues with non-zero expression level. GO classes belong to the plant GO slim subset. Ontology: CC, cellular component; BP, biological process; MF: molecular function.

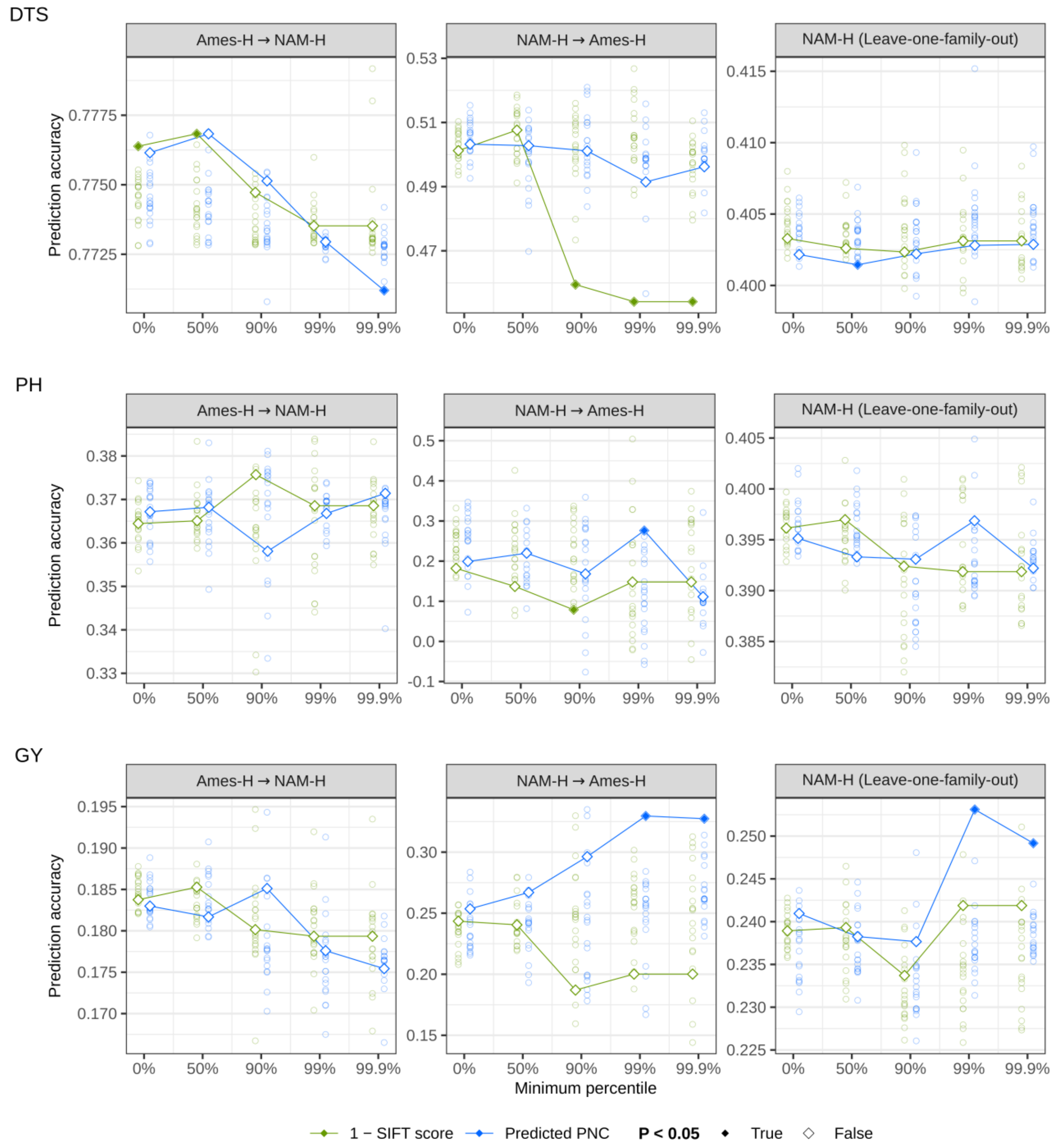

Fig. S8 Prioritization of nonsynonymous SNPs in genomic prediction for agronomic traits in hybrid panels. Agronomic traits: days to silking (DTS), plant height (PH) and grain yield (GY); hybrid panels: Nested Association Mapping hybrid panel (NAM-H), diverse hybrid panel (Ames-H) [16]. Genomic prediction accuracy was estimated within NAM-H (in leave-one-family-out prediction), from NAM-H to Ames-H, and from Ames-H to NAM-H. Genomic prediction models included effects of population structure variables (top three principal components from the Hapmap 3.2.1 reference panel in maize),

effects of genome-wide SNPs, and effects of nonsynonymous SNPs. Diamonds: nonsynonymous SNPs were weighted by SIFT conservation (1 − SIFT score) or predicted PNC, and prioritized by truncating weights to zero if they were under the 0%, 50%, 90%, 99%, or 99.9% percentile. Open circles: nonsynonymous SNPs were weighted and prioritized by 20 random permutations of SIFT conservation or predicted PNC, to determine whether the prediction accuracy by SNP weights was significantly different from the accuracy by random SNP weights.
