# Supplementary material for: Prediction of evolutionary constraint by genomic annotations improves functional prioritization of genomic variants in maize
Source: Genome Biol. 2022 Sep 1;23:183. doi: 10.1186/s13059-022-02747-2 (PMC9438327; doi:10.1186/s13059-022-02747-2)
Supplement: Supplementary file 3 — Additional file 3. Review history. [file 13059_2022_2747_MOESM3_ESM.docx]

Review History

**First round of review**

**Reviewer 1**

**Were you able to assess all statistics in the manuscript, including the appropriateness of statistical tests used?**

No, I do not feel adequately qualified to assess the statistics.

**Comments to author:**

The overarching topic of the manuscript of Ramstein and Buckler, using phylogenetic sequence conservation to perform genomic prediction model used in agricultural genetics, is very timely and of interest to a diverse audience of plant breeders and geneticists. But the presentation of the results does not serve this goal. The manuscript is written in a highly technical manner that is better suited to a journal focusing on theoretical results in population or quantitative genetics and makes to many assumptions of prior knowledge on part of the reader. In addition to a thorough rewrite, the manuscript will profit from the application of the authors' analytical framework to more crops, including inbreeding and outcrossing taxa, and the proper documentation of their code. The code deposited in the cited Bitbucket repository enables reproducing the results obtained by the authors, but I think they should have set themselves the more ambitious goal of making their pipeline easily accessible to the general computational plant geneticist to bring about the application of the phylogenetically informed genomic prediction heralded in the abstract.

In the following, I point out instance where the reading was tough for me.

ll. 15-16: The statement is logically correct, but it is only genome editing that requires single-bp resolution

l. 20: I believe Angiosperms need not be capitalized, see for example https://doi.org/10.1038/s41477-019-0421-0

l. 46: The causative nature of mutations cannot be established by phylogenetic analysis, but only by functional analysis of natural and induced mutants.

ll. 54-56: This is a highly technical way of saying that even sites at which mutation exert moderate effects on fitness are highly conserved between species.

ll. 58-60 What is a specific example of a genomic annotation that captures the fitness effects. Genomic annotation commonly refers to structural and functional gene annotations neither of which implies fitness effects.

l. 68: My understanding is that annotations can have missing data, e.g. missed gene models due to lack of transcript evidence, novel genes, or functional annotation that say "protein of unknown function"

l. 76: "Large"without more quantification does not mean anything. For example, the evolutionary with maize and teosinte may be prohibitively large for a plant breeder.

ll. 91-92: the term "rejected substitution" needs more explanation. Why was this terminology chosen?

ll. 95-96: I was unable to follow the logic of this sentence. If a site is monomorphic, there are no mutations, non-synonymous or the other kind.

ll. 97: I believe to grasp the concept of survivorship bias. But I was not able to complete the logical chain linking the previous sentence to the avoidance of that type of bias.

ll. 100: Describing Hapmap 3.2.1. as *the* reference panel presuppose a familiarity with the current state of maize genomics beyond that of this reviewer.

ll. 112: SIFT and UniRep are in need of definition. A clear motivation needs to be given why these datasets/methods were chosen for a non-technical audience.

ll. 113-114: The description of item (iii) is too terse.

ll. 120: novel: What is new about the UniRep annotations, do you simply mean "more" or "more complete"?

ll. 131: Which properties of genes do you have in mind?

ll. 138: A function is said to be "smooth" if it can be differentiated infinitely many times. My understanding of mathematics is that for this to be meaningful the functions domain need to a complete vector space such as the real numbers, but genomic annotations are discrete and countable.

ll. 150: "only for observed PNC" seems to convey a sense of disappointment, but it's not clear in which other regards a significant increase should have been expected in this context.

ll. 186: The last sentence seems to conclude that hypothesis put forward in ll. 156-158, namely that PNC identifies "impactful" genes, cannot be rejected. I gather from the intervening paragraph that PNC fails to highlight developmental genes, which should been there. So maybe the hypothesis does have to rejected, after all?

ll. 207: "purging of mutation load" Is this a mere theoretical possibility / scientific vision or a goal attainable in next couple of decades? I expect there are review or perspective article elaborating on this matter that the authors could cite.

ll. 209: Again, introducing NAM-H as a panel of half-sib presupposes too much familiarity with maize genetics.

ll. 237: negative selection: what about balancing selection. This may play a role in disease resistance, a trait cherished by breeders

ll. 281-282: "coming generation" Is this an opaque reference to computational prediction of protein folding by a subsidiary of the company Alphabet? I believe there was a paper on that method that should be cited.

**Reviewer 2**

**Were you able to assess all statistics in the manuscript, including the appropriateness of statistical tests used?**

No, I do not feel adequately qualified to assess the statistics.

**Comments to author:**

This paper describes a new procedure (PICNC) to identify SNPs with possibly large fitness effects based on phylogenetic conservation. The authors then examine their method in the context of maize mapping and fitness.

This is an important paper that pushes a continuing trend in the field to see if we can identify mutations with fitness effects. This is key in various field - not only for genome annotation, but also applied to human disease mapping and in their case agronomic applications.

I cannot comment on the core of the method itself (other than to say the logic seems good) but I will focus on some minor issues that I think the authors need to add to their text to help readers along.

1. I was not clear here what they mean by monomorphic site - did they mean no SNPs in maize? Or no nucleotide differences in the MSA (but could have SNPs in maize)? Maybe defining this early in the text could help.

2. The authors immediately jump to their MSA, but it would be good to tell the reader in the main text what the species are in the MSA (all angiosperms, within Poaceae, etc.) I know that one can glean this by looking at the figure but it would help the readers if they just describe the MSA.

3. I am surprised at the really low level of deleterious mutations they think they identify per maize individual - 15 seems low? Maybe they could add more context in terms of what other studies have shown and also the possibility that much of the fitness loss is polygenic with lots of mutations of very slightly deleterious effects? In other words, what fraction of fitness burden do these 15 represent vs total deleterious load.

4. I take it that because their method appears to focus on nonsynonymous mutations they cannot apply this to intergenic, or noncoding regions? Is this correct? If so a discussion of this limitation and what it means with respect to deleterious burden may be interesting (that is, are most deleterious mutants in proteins rather than cis-regulatory elements)?

**Reviewer 3**

**Were you able to assess all statistics in the manuscript, including the appropriateness of statistical tests used?**

Yes, and I have assessed the statistics in my report.

`

**Comments to author:**

Evolutionary constraint refers to the factors that limit the action of natural selection, and then phenotypic changes will not occur at random. Thus, genes featuring high degree of evolutionary constraints tend not to change and usually maintain high conservation in sequences and functions across different species. In population genetics, quantitative assessments of evolutionary constraints are mostly based on statistical analysis. In this work, the authors present a machine learning (ML)-based method, the Random Forest (RF) algorithm, to predict the level of evolutionary constraints occurred on maize genes. They constructed a library of predictors or features, including SIFT score, GC content, K-mer, TE insertion, protein structures etc. Based on these predictors, the RF model predicts the degree of constraints on the ~25000 maize genes, which is defined by an index called PNC (phylogenetic nucleotide conservation). The higher of PNC, the higher degree of evolutionary constraints.

Then, the authors demonstrate the usability of PNC that can be used as an indicator that may help identify the causal variants affecting gene functions. This application has to be based on the assumption that genes targeted by artificial selection, at least part of genes utilized in breeding, are more evolutionarily conserved and functionally constrained than those genes that are not targeted by selection. To verify this assumption, the authors performed genotype-to-phenotype (G2P) prediction using a mixed linear model (MLM) integrated with PNCs to weight the effects of SNPs. This strategy is essentially a way of filtering SNPs, as it may reduce the total of 104,000 SNPs to 1040 SNPs (top 1%) and 104 SNPs (top 0.1%). Based on the result that prediction accuracies of grain yield when using 1040 and 104 SNPs are slightly higher than that when using of 104,000 SNPs, they believe their rationales are validated.

This work is potentially useful for certain aspects. For instance, it helps estimate the degree of evolutionary constraint of a SNP and functional conservation of a gene; and it helps identify potentially causal variants occurred in the coding sequences from GWAS result. However, the authors need to first rule out any possibility of model overfitting, especially when the information of genomic annotations are added as features, although these features improve predictability from 0.72 to 0.80. The current validation scheme is still a self-validation, and it still needs a lot of work to make sure that validation is objective. Also, there are major flaws in the part of genomic prediction and thus the results from G2P prediction may not support the assumption. My major concerns are described below.

1. The main selling point of this work is to present a novel ML-based methodology to estimate evolutionary constraints of genes. However, any possibility of causing overfitting has to be ruled out. Additionally, the authors' basic assumption is that DNA sites featuring high conservation tend to be targeted by artificial selection. Since, there are a handful of genes in maize have been reported involved in domestication selection, photoperiodic adaptation selection (such as, flowering-time genes), and trait improvement selection. I am wondering how many of them and SNP sites are among the list of 90%, 95%, 99% and 99.9% PNC percentiles. If the authors can give out some examples of selected genes, it would be very convincing.

Additionally, if the authors only performed within-species validation using maize genomic annotation and SIFT scores. The leave-one-chromosome-out scheme may rule out some of the positional influence as observed sites and predicted site may share the same genomic context. However, if the authors' basic assumption is correct in maize, it could be applied to all crop species, like rice and soybean. There are plenty of genomic and population data that the users may be used to validate their assumption. If I understand correctly on the procedure of predicting PNC, between-species validation only using SIFT scores as predictors is doable. I highly encourage the authors to design a validation scheme that may use the model trained in maize to predict PNC in rice. If the predictability maintained at 70% or even lower, it will be very convincing.

2. Why the authors choose the RF algorithm as predictive model. There are many better models, such as tree-based LightGBM, XGBoost etc. which are more robustness and efficient than RFs. The authors should perform model evaluation and select the optimal one. Additionally, the authors should include optimal hyper-parameters determined by grid search-based model tuning.

3. The SIFT scores ranging from 0.0 (deleterious) to 1.0 (tolerated), which is also developed by the author's lab to predict whether an amino acid substitution affects protein function based on sequence alignments across different species, likely contribute the most when predicting PNC. Is there any correlation between SIFT and PNC? The SNPs at the 99% and 99.9% quantile show higher or lower SIFT score? Whether the SIFT score can be directly used as weights in the MLM model to predict phenotype?

4. According to Figure 4 and 5, mRNA and proteins of the genes with high PNC (99.9% percentile) tend to express in multiple tissues and high abundance. This result indicates that they are probably housekeeping genes. However, my knowledge of the genes selected by breeding is against this result. The genes targeted by breeding selection are likely non-essential genes, and genetic variations benefiting human-desired trait will not influence the normal development of a plant. The functional enrichment analysis of high-PNC genes is too coarse.

5. This method could be potentially used to identify causal genes and variants from the GWAS results, as the majority of SNPs significantly associated with an agronomic trait are linked SNPs within a QTL. Is it possible to use the PNC to prioritize the causality of genes to short the candidate list and help identify the causal variants for further functional validation and design of genome editing sites? Multiple previous research in plants and animals have utilized genomic features and functional annotations in a ML system to prioritize genes from GWAS results. The authors may follow these works and it would be a big help if this ideal is successfully implemented.

6. Genomic prediction is the most problematic part. However, this is the most important part, because the authors want to use this result to support their assumption and indicate the usability of the SNPs in breeding. A recent study systematically evaluated 12 ML methods in comparison with rrBLUP for predicting three agronomic traits (flowering time, plant height and grain yield) in six plant species (Azodi et al., 2019). They finally concluded that "No single method performed best across all species and traits, and model tuning for automated optimization of hyper-parameters using grid search was the key to achieving the best performance for all ML methods." If the author wants to include genomic prediction, they have to follow the standard procedure of model evaluation using multiple traits and population.

According to Figure 4a, the minor allele frequency (MAF) of SNPs featuring high PNC are mostly below 0.05 or even close to 0. These low-MAF SNPs are usually removed from G2P prediction, as the chance of no polymorphism occurred in training and testing population is too high. I also think the slight elevation of accuracy of predicting grain yield is likely attributed to the low MAF SNPs filtered by PNC. Additionally, to ensure the improvement is not a coincidence, the authors need to compile a set of randomly selected SNPs with equal amount of SNPs in different PNC percentile. It has to be done by 100 times of randomization and plot a distribution and include significance test of the comparison.

Azodi, C.B., Bolger, E., McCarren, A., Roantree, M., de Los Campos, G. and Shiu, S.H. (2019) Benchmarking Parametric and Machine Learning Models for Genomic Prediction of Complex Traits. G3 (Bethesda), 9, 3691-3702

7. Line 88, "evolutionary constraint (negative selection)". Strictly speaking, evolutionary constraint is not equal to negative selection. Negative selection is to remove deleterious alleles from a population, and evolutionary constraint describe the functional importance of a site. Because the site is too important, it doesn't allow mutations occurred on this site.

**Authors Response**

**Point-by-point responses to the reviewers’ comments:**

Reviewer #1:

The overarching topic of the manuscript of Ramstein and Buckler, using phylogenetic sequence conservation to perform genomic prediction model used in agricultural genetics, is very timely and of interest to a diverse audience of plant breeders and geneticists. But the presentation of the results does not serve this goal. The manuscript is written in a highly technical manner that is better suited to a journal focusing on theoretical results in population or quantitative genetics and makes to many assumptions of prior knowledge on part of the reader.

*Reply: We addressed each of the reviewer’s comments regarding writing style and clarity (see responses below). We also made other edits throughout the manuscript, to make it more accessible to a larger audience (see tracked changes).*

In addition to a thorough rewrite, the manuscript will profit from the application of the authors' analytical framework to more crops, including inbreeding and outcrossing taxa, and the proper documentation of their code.

*Reply: In addition to our initial results in maize, we added results in sorghum. We trained our model on the whole maize genome. Then, we produced genomic annotations in the sorghum reference genome, and predicted PNC in sorghum, based on the model trained in maize. Additional validations based on minor allele frequency in sorghum suggest that our model is capable of prioritizing loci under negative selection, even in a different genome, in a taxon that is predominantly selfing, unlike maize (predominantly outcrossing). We added paragraphs in the Results section (ll. 210-229) and Methods section (ll. 531-549), as well as two figures (Fig. 5 and S6), to present these new results.*

The code deposited in the cited Bitbucket repository enables reproducing the results obtained by the authors, but I think they should have set themselves the more ambitious goal of making their pipeline easily accessible to the general computational plant geneticist to bring about the application of the phylogenetically informed genomic prediction heralded in the abstract.

*Reply: We deposited our data in CyVerse (*[*https://doi.org/10.25739/hybz-2957*](https://doi.org/10.25739/hybz-2957)*). To make it clear that this data was made publicly available, we added a sentence in the end of the abstract, which contained the link to the data and described its content (“trained PICNC models and predicted nucleotide conservation at protein-coding SNPs in maize”). Compared to the previous version of the manuscript, this dataset now contains the whole-genome fit in maize, which can be used to make prediction in other genomes than maize. It also contains all features (genomic annotations and predicted PNC, at SNPs and genes) and the leave-one-chromosome-out fits of the PICNC model in maize.*

In the following, I point out instance where the reading was tough for me.

ll. 15-16: The statement is logically correct, but it is only genome editing that requires single-bp resolution.

*Reply: In the abstract, we changed the mention “single-site resolution” to “high resolution, within fewer than hundreds base pairs”. Moreover, in the ‘Background’ section, we added the following mention: “identification of candidate causal mutations at high resolution: within hundreds of base pairs for cross-population genomic prediction or gene knock-out, and at single-base resolution for CRISPR-based editing”.*

l. 20: I believe Angiosperms need not be capitalized, see for example <https://doi.org/10.1038/s41477-019-0421-0>.

*Reply: We removed the capitalization at Angiosperms.*

l. 46: The causative nature of mutations cannot be established by phylogenetic analysis, but only by functional analysis of natural and induced mutants.

*Reply: We agree that statistical analyses (analyses of association or conservation) can only point to candidate mutations. Throughout the manuscript, we changed “causal mutations” to “candidate causal mutation” whenever they referred to inferences from statistical analyses. We also changed the title of the manuscript to better reflect the distinction pointed out by the reviewer: “Prediction of evolutionary constraint by genomic annotations improves functional prioritization of genomic variants in maize”.*

ll. 54-56: This is a highly technical way of saying that even sites at which mutation exert moderate effects on fitness are highly conserved between species.

*Reply: We changed the explanation to “[observed PNC] may lack discriminatory power, because even variants with moderate effect on fitness may be highly conserved”. We changed the term sensitivity (which may be ambiguous in the context of classification) to “discriminatory power”, in the ‘Background’ and ‘Discussion’ sections.*

ll. 58-60 What is a specific example of a genomic annotation that captures the fitness effects. Genomic annotation commonly refers to structural and functional gene annotations neither of which implies fitness effects.

*Reply: We added examples of genomic annotations used to predict fitness effects, from the citations mentioned in this sentence: “epigenetic marks, amino acid change, or disruption of transcription factor motifs”.*

l. 68: My understanding is that annotations can have missing data, e.g. missed gene models due to lack of transcript evidence, novel genes, or functional annotation that say "protein of unknown function".

*Reply: Our genomic annotations did not rely directly on any experimental data. For example, we did not use GO annotations to predict PNC, because these features ultimately point to experimentally validated gene functions, through sequence homology, and may be missing. However, we agree that these computational annotations require gene-model annotations. Therefore, we added the mention that our computational annotations “are readily available from DNA sequence data and gene-model annotations”.*

l. 76: "Large"without more quantification does not mean anything. For example, the evolutionary with maize and teosinte may be prohibitively large for a plant breeder.

*Reply: To be more specific about the timescale of PNC, we replace “at large evolutionary timescale” with “in the angiosperm clade, spanning > 100 million years of evolution and recombination events”, based on estimates of Chaw et al. (2004;* [*http://dx.doi.org/10.1007/s00239-003-2564-9*](http://dx.doi.org/10.1007/s00239-003-2564-9)*).*

ll. 91-92: the term "rejected substitution" needs more explanation. Why was this terminology chosen?

*Reply: ‘Rejected substitution’ is the term used by the authors of the gerp++ software, to refer to their metric for constrained evolution (Davydov et al. 2010). In the main text, we changed the mention “their ‘rejected substitutions’ (RS) scores were higher” to “their evolutionary rate was lower” so that the sentence is more readable. However, we have kept the description of RS scores, in the legend of Fig. 1, which is more detailed.*

ll. 95-96: I was unable to follow the logic of this sentence. If a site is monomorphic, there are no mutations, non-synonymous or the other kind.

*Reply: We changed this sentence to “PICNC did not rely on within-species variability (e.g., SNP allele frequency), so we could train our model on nonsynonymous mutations at any site, even when they were not observed in maize populations (i.e., even when they were monomorphic within maize)”. In our approach, we do not need to observe a mutation in any maize population, because we do not need allele-frequency information to make inferences about fitness effects.*

ll. 97: I believe to grasp the concept of survivorship bias. But I was not able to complete the logical chain linking the previous sentence to the avoidance of that type of bias.

*Reply: To clarify the link between the previous sentence and survivorship bias, we added the sentence “Therefore, we could include data about the genomic sites where polymorphisms are not tolerated by evolution (e.g., the sites where mutations are lethal)”. We hope that this additional sentence makes it clearer that survivorship bias refers to the overrepresentation of tolerated mutations in SNP data.*

ll. 100: Describing Hapmap 3.2.1. as *the* reference panel presuppose a familiarity with the current state of maize genomics beyond that of this reviewer.

*Reply: We changed the mention of Hapmap 3.2.1 to a more general term (“across diverse maize lines”) while keeping the reference about this panel (Bukowsky et al. 2018). Throughout the main text, we also avoided the mention of Hapmap 3.2.1 to avoid confusion among readers who are not familiar to maize genetic resources.*

ll. 112: SIFT and UniRep are in need of definition. A clear motivation needs to be given why these datasets/methods were chosen for a non-technical audience.

*Reply: We added the following sentences:*

*“While mutation type simply characterizes the codon change caused by a nonsynonymous mutation (missense, STOP gain or STOP loss), SIFT score quantifies the impact of the nonsynonymous mutation by calculating the probability of its codon change (the lower the SIFT score, the more damaging the mutation) [3,16]. In contrast, UniRep variables characterize the ontology and structure of a protein by a quantitative representation of its sequence [13]; in silico mutagenesis scores then quantify the effect of a mutation on this representation. Therefore, mutation type, SIFT score, and in silico mutagenesis scores all measured how damaging a codon change was, but in different ways.” (ll. 130-138)*

*We hope that this addition will help the readers understand that UniRep is complementary to mutation type and SIFT score, in that it provides a radically new way of characterizing the damaging impact of mutations.*

ll. 113-114: The description of item (iii) is too terse.

*Reply: We added the following sentences, to describe leave-one-chromosome-out prediction and its importance for avoiding spurious patterns:*

*“For each of the ten chromosomes in maize, we predicted PNC using a model trained in all other chromosomes. Therefore, the accuracy of our approach could not be inflated by spurious associations between genomic annotations and PNC along chromosomes.”*

ll. 120: novel: What is new about the UniRep annotations, do you simply mean "more" or "more complete"?

*Reply: We added the mention that genomic annotations from UniRep are “based on machine learning techniques (protein embedding) rather than evolutionary or bioinformatics approaches”. Combined with additional explanations of mutation type, SIFT score, and UniRep variables (ll. 130-138), we hope that this mention makes it clearer that UniRep is different from other annotations, because it is based on different tools, to produce a quantitative representation of protein variability.*

ll. 131: Which properties of genes do you have in mind?

*Reply: We rewrote the sentence to make it clearer that the gene properties were “expression levels (RNA and protein abundance) and selective constraint (negatively associated with the nonsynonymous-to-synonymous SNP ratio, Pn/Ps, and the proportion of nonsynonymous SNPs, Pn/(Pn+Ps), within each gene)”.*

ll. 138: A function is said to be "smooth" if it can be differentiated infinitely many times. My understanding of mathematics is that for this to be meaningful the functions domain need to a complete vector space such as the real numbers, but genomic annotations are discrete and countable.

*Reply: Here “smooth” was not used in this sense, but we agree that “smooth” should refer to the mathematical property of infinite differentiability. Therefore, we removed this mention and wrote instead that “functions of genomic annotations [were] learned across many sites, and predicting a quantitative value for the probability of PNC, ranging between 0 and 1”.*

ll. 150: "only for observed PNC" seems to convey a sense of disappointment, but it's not clear in which other regards a significant increase should have been expected in this context.

*Reply: We agree that this lack of association does not necessarily reveal a shortcoming of the approach. We rewrote the sentence to avoid the negative connotation: “a positive association with expression QTL (eQTL) effect was also observed, but only for observed PNC”.*

ll. 186: The last sentence seems to conclude that hypothesis put forward in ll. 156-158, namely that PNC identifies "impactful" genes, cannot be rejected. I gather from the intervening paragraph that PNC fails to highlight developmental genes, which should been there. So maybe the hypothesis does have to rejected, after all?

*Reply: To further investigate functional enrichments by our gene prioritizations, we conducted a pathway enrichment analysis (using annotations from CornCyc). We added a paragraph in the Results section (ll. 267-278) and a table (Table 1) to show that genes involved in central carbon metabolism tend to be prioritized more. We also added a paragraph in the Discussion section (ll. 363-390) to discuss the limitations of our approach. In particular, we explained that “the emphasis of predicted PNC on primary metabolism illustrates a fundamental trade-off in our approach (and other similar approaches like SIFT): on the one hand, evolutionary depth allows us to detect effects of mutations at high spatial resolution; on the other hand, inferences are biased towards certain categories of genes, because fitness effects can only be detected if they are consistent over large evolutionary timescales”. In the same paragraph, we also explained that predicted PNC is nonetheless useful to detect some of the genes and mutations that have high impact on fitness-related traits.*

ll. 207: "purging of mutation load" Is this a mere theoretical possibility / scientific vision or a goal attainable in next couple of decades? I expect there are review or perspective article elaborating on this matter that the authors could cite.

*Reply: ‘Purging of mutational load’ refers to methods which already exist but are not yet broadly implemented, for removing deleterious mutations: rapid cycling genomic selection, or targeted mutagenesis (multiplexed CRISPR-based base editing). We added a paragraph in the Discussion section (ll. 363-390) to mention these applications, and cite important reviews on the topic: Varshney et al. (2021), Molla et al. (2021) and Gao (2021).*

ll. 209: Again, introducing NAM-H as a panel of half-sib presupposes too much familiarity with maize genetics.

*Reply: We added a brief explanation about the half-sib family structure, “in which 24 different donor parents are crossed to the same recurrent parent”, and added a citation about this panel (Ramstein et al. 2020). We chose not to remove the mention of the half-sib family structure, because it is such an important feature of this panel, but we do hope that this additional explanation will make it less cryptic to a broad audience.*

ll. 237: negative selection: what about balancing selection. This may play a role in disease resistance, a trait cherished by breeders.

*Reply: We added a paragraph in the Discussion section (ll. 363-390) to address this limitation. We cite relevant literature (Moyers et al. 2018, Jacobowitz et al. 2020, Butt et al. 2020) and explain that mutations impacting genes in specialized metabolism are more likely to be detected by other approaches: “analysis of evolutionary divergence or balancing selection, association studies and/or targeted mutagenesis”. As pointed out by Moyers et al. (2018), phylogenetic approaches (in general) lack power to detect clade- or environment-specific fitness effects. We discuss this caveat, and alternate approaches, in the same paragraph.*

ll. 281-282: "coming generation" Is this an opaque reference to computational prediction of protein folding by a subsidiary of the company Alphabet? I believe there was a paper on that method that should be cited.

*Reply: We added a sentence to describe and provide citations about two types of protein structural annotations which will be useful to predict PNC: protein representation (e.g., UniRep, as well as other techniques) and 3D protein structure (e.g., AlphaFold 2, which is produced by DeepMind, indeed a subsidiary of Alphabet).*

*References:*

*Butt, H., Zaidi, S. S.-E.-A., Hassan, N., & Mahfouz, M. (2020). CRISPR-Based Directed Evolution for Crop Improvement. Trends in Biotechnology, 38(3), 236–240.* [*https://doi.org/10.1016/j.tibtech.2019.08.001*](https://doi.org/10.1016/j.tibtech.2019.08.001)

*Chaw, S.-M., Chang, C.-C., Chen, H.-L., & Li, W.-H. (2004). Dating the monocot-dicot divergence and the origin of core eudicots using whole chloroplast genomes. Journal of Molecular Evolution, 58(4), 424–441.* [*https://doi.org/10.1007/s00239-003-2564-9*](https://doi.org/10.1007/s00239-003-2564-9)

*Davydov, E. V., Goode, D. L., Sirota, M., Cooper, G. M., Sidow, A., & Batzoglou, S. (2010). Identifying a high fraction of the human genome to be under selective constraint using GERP++. PLoS Computational Biology, 6(12), e1001025.* [*https://doi.org/10.1371/journal.pcbi.1001025*](https://doi.org/10.1371/journal.pcbi.1001025)

*Gao, C. (2021). Genome engineering for crop improvement and future agriculture. Cell.* [*https://doi.org/10.1016/j.cell.2021.01.005*](https://doi.org/10.1016/j.cell.2021.01.005)

*Jacobowitz, J. R., & Weng, J.-K. (2020). Exploring Uncharted Territories of Plant Specialized Metabolism in the Postgenomic Era. Annual Review of Plant Biology, 71, 631–658.* [*https://doi.org/10.1146/annurev-arplant-081519-035634*](https://doi.org/10.1146/annurev-arplant-081519-035634)

*Molla, K. A., Sretenovic, S., Bansal, K. C., & Qi, Y. (2021). Precise plant genome editing using base editors and prime editors. Nature Plants, 1–22.* [*https://doi.org/10.1038/s41477-021-00991-1*](https://doi.org/10.1038/s41477-021-00991-1)

*Moyers, B. T., Morrell, P. L., & McKay, J. K. (2018). Genetic Costs of Domestication and Improvement. The Journal of Heredity, 109(2), 103–116.* [*https://doi.org/10.1093/jhered/esx069*](https://doi.org/10.1093/jhered/esx069)

*Ramstein, G. P., Larsson, S. J., Cook, J. P., Edwards, J. W., Ersoz, E. S., Flint-Garcia, S., Gardner, C. A., Holland, J. B., Lorenz, A. J., McMullen, M. D., Millard, M. J., Rocheford, T. R., Tuinstra, M. R., Bradbury, P. J., Buckler, E. S., & Romay, M. C. (2020). Dominance Effects and Functional Enrichments Improve Prediction of Agronomic Traits in Hybrid Maize. Genetics, 215(1), 215–230.* [*https://doi.org/10.1534/genetics.120.303025*](https://doi.org/10.1534/genetics.120.303025)

*Varshney, R. K., Bohra, A., Yu, J., Graner, A., Zhang, Q., & Sorrells, M. E. (2021). Designing Future Crops: Genomics-Assisted Breeding Comes of Age. Trends in Plant Science, 26(6), 631–649.* [*https://doi.org/10.1016/j.tplants.2021.03.010*](https://doi.org/10.1016/j.tplants.2021.03.010)

Reviewer #2:

This paper describes a new procedure (PICNC) to identify SNPs with possibly large fitness effects based on phylogenetic conservation. The authors then examine their method in the context of maize mapping and fitness.

This is an important paper that pushes a continuing trend in the field to see if we can identify mutations with fitness effects. This is key in various field - not only for genome annotation, but also applied to human disease mapping and in their case agronomic applications.

I cannot comment on the core of the method itself (other than to say the logic seems good) but I will focus on some minor issues that I think the authors need to add to their text to help readers along.

1. I was not clear here what they mean by monomorphic site - did they mean no SNPs in maize? Or no nucleotide differences in the MSA (but could have SNPs in maize)? Maybe defining this early in the text could help.

*Reply: In the first subsection of the Results section (ll. 103-109), we added a short description of monomorphic sites (“sites with no observed SNPs in a maize reference panel “). We also added the following sentences to explain that we included nonsynonymous mutations which were not necessarily observed (as SNPs) in maize populations: “PICNC did not rely on within-species variability (e.g., SNP allele frequency), so we could train our model on nonsynonymous mutations at any site, even when they were not observed in maize populations (i.e., even when they were monomorphic within maize). Therefore, we could include data about the genomic sites where polymorphisms are not tolerated by evolution (e.g., the sites where mutations are lethal)” (ll. 112-117).*

2. The authors immediately jump to their MSA, but it would be good to tell the reader in the main text what the species are in the MSA (all angiosperms, within Poaceae, etc.) I know that one can glean this by looking at the figure but it would help the readers if they just describe the MSA.

*Reply: We added a paragraph in the Results section (ll. 91-102), and described the MSA by the following sentences: “PNC was estimated by conservation of DNA bases, in a MSA across 27 diverse plant genomes, from dicots (Trifolium pratense, Medicago truncatula, Glycine max, Prunus persica, Populus trichocarpa, Manihot esculenta, Arabidopsis thaliana, Arabidopsis lyrata, Brassica napus, Brassica rapa, Theobroma cacao, Vitis vinifera, Solanum tuberosum, Solanum lycopersicum, Chenopodium quinoa, Beta vulgaris) to monocots (Sorghum bicolor, Setaria italica, Oryza rufipogon, Oryza longistaminata, Leersia perrieri, Triticum Urartu, Aegilops tauschii, Hordeum vulgare, Brachypodium distachyon, Musa acuminata, and Amborella trichopoda) [14]. Consequently, this MSA spanned large evolutionary times, equivalent to 16.2 expected substitutions per site under a neutral evolutionary model”.*

3. I am surprised at the really low level of deleterious mutations they think they identify per maize individual - 15 seems low? Maybe they could add more context in terms of what other studies have shown and also the possibility that much of the fitness loss is polygenic with lots of mutations of very slightly deleterious effects? In other words, what fraction of fitness burden do these 15 represent vs total deleterious load.

*Reply: This small number of mutations is the number of segregating variants having a predicted PNC above the 99.9%-percentile. The 99.9%-percentile threshold is arbitrary, so it should not be used to distinguish putatively deleterious mutations from putatively non-deleterious mutations. Instead, we suggest that this small set of prioritized SNPs could represent a useful set of targets for purging mutational load, since they are the most impactful mutations according to predicted PNC. To convey the arbitrary nature of this threshold, and to give an estimate of the fraction of the mutational load that these few selected SNPs represent, we added the following sentences in the Result section: “Such few selected variants would represent a small fraction of the total mutational load, since even most stringent prioritizations by SIFT scores would select as many as 1638 deleterious mutations per inbred line on average (Table S1). However, they would represent the fraction of most impactful SNPs in each haploid genome” (ll. 302-306).*

4. I take it that because their method appears to focus on nonsynonymous mutations they cannot apply this to intergenic, or noncoding regions? Is this correct? If so a discussion of this limitation and what it means with respect to deleterious burden may be interesting (that is, are most deleterious mutants in proteins rather than cis-regulatory elements)?

*Reply: It is correct that our method should not be applied to non-coding and intergenic regions, because some useful genomic annotations cannot describe the impact of mutations in these regions (e.g., mutation type, SIFT score, and UniRep in silico mutagenesis scores). Importantly, UniRep variables did not improve accuracy for predicted PNC in these regions. In the Results section, we added a paragraph (ll. 183-189) and a figure (Fig. S5) to present this result and conclude that “while UniRep variables may be useful to predict the effects of mutations on protein structure, they may not provide useful information for effects related to other biological processes (e.g., regulation of gene expression)”. In the Discussion section, we added the following sentences to explain that UniRep variables should not be used outside protein-coding regions: “the genomic annotations produced by UniRep described protein structural variation, using only protein sequence information [13]. As expected, these annotations were useful to describe fitness effects of nonsynonymous mutations, but they were not useful to predict the impact of mutations in UTRs, introns, or intergenic regions (Supplementary Fig. S5). Importantly, UniRep features actually decreased the accuracy of predicted PNC for mutations in these regions. Therefore, different sets of computational annotations should probably be selected to predict PNC at different categories of DNA sites” (ll. 422-429).*

Reviewer #3:

Evolutionary constraint refers to the factors that limit the action of natural selection, and then phenotypic changes will not occur at random. Thus, genes featuring high degree of evolutionary constraints tend not to change and usually maintain high conservation in sequences and functions across different species. In population genetics, quantitative assessments of evolutionary constraints are mostly based on statistical analysis. In this work, the authors present a machine learning (ML)-based method, the Random Forest (RF) algorithm, to predict the level of evolutionary constraints occurred on maize genes. They constructed a library of predictors or features, including SIFT score, GC content, K-mer, TE insertion, protein structures etc. Based on these predictors, the RF model predicts the degree of constraints on the ~25000 maize genes, which is defined by an index called PNC (phylogenetic nucleotide conservation). The higher of PNC, the higher degree of evolutionary constraints.

Then, the authors demonstrate the usability of PNC that can be used as an indicator that may help identify the causal variants affecting gene functions. This application has to be based on the assumption that genes targeted by artificial selection, at least part of genes utilized in breeding, are more evolutionarily conserved and functionally constrained than those genes that are not targeted by selection. To verify this assumption, the authors performed genotype-to-phenotype (G2P) prediction using a mixed linear model (MLM) integrated with PNCs to weight the effects of SNPs. This strategy is essentially a way of filtering SNPs, as it may reduce the total of 104,000 SNPs to 1040 SNPs (top 1%) and 104 SNPs (top 0.1%). Based on the result that prediction accuracies of grain yield when using 1040 and 104 SNPs are slightly higher than that when using of 104,000 SNPs, they believe their rationales are validated.

This work is potentially useful for certain aspects. For instance, it helps estimate the degree of evolutionary constraint of a SNP and functional conservation of a gene; and it helps identify potentially causal variants occurred in the coding sequences from GWAS result. However, the authors need to first rule out any possibility of model overfitting, especially when the information of genomic annotations are added as features, although these features improve predictability from 0.72 to 0.80. The current validation scheme is still a self-validation, and it still needs a lot of work to make sure that validation is objective. Also, there are major flaws in the part of genomic prediction and thus the results from G2P prediction may not support the assumption. My major concerns are described below.

1. The main selling point of this work is to present a novel ML-based methodology to estimate evolutionary constraints of genes. However, any possibility of causing overfitting has to be ruled out. Additionally, the authors' basic assumption is that DNA sites featuring high conservation tend to be targeted by artificial selection. Since, there are a handful of genes in maize have been reported involved in domestication selection, photoperiodic adaptation selection (such as, flowering-time genes), and trait improvement selection. I am wondering how many of them and SNP sites are among the list of 90%, 95%, 99% and 99.9% PNC percentiles. If the authors can give out some examples of selected genes, it would be very convincing.

*Reply: To characterize functional enrichments by our gene prioritizations, we conducted a pathway enrichment analysis (using annotations from CornCyc) and we made a supplementary file (File S1) in which we list the genes prioritized by different measures of fitness effects (predicted/observed PNC, SIFT score), at different thresholds. We added a paragraph in the Results section (ll. 267-278) and a table (Table 1), in which we showed that genes involved in central carbon metabolism tend to be prioritized more. We also added a paragraph in the Discussion section (ll. 363-390) to discuss the limitations of our approach. In particular, we explained that “the emphasis of predicted PNC on primary metabolism illustrates a fundamental trade-off in our approach (and other similar approaches like SIFT): on the one hand, evolutionary depth allows us to detect effects of mutations at high spatial resolution; on the other hand, inferences are biased towards certain categories of genes, because fitness effects can only be detected if they are consistent over large evolutionary timescales”.*

Additionally, if the authors only performed within-species validation using maize genomic annotation and SIFT scores.

*Reply: In the Results section (subsection “Predicted evolutionary constraint identifies deleterious variants”), we added one paragraph (ll. 210-229) and a Figure (Fig. 5) to compare SIFT score and predicted PNC for average decrease in MAF. We showed that prioritizations by SIFT score result in a smaller decrease in MAF, compared to predicted PNC.*

The leave-one-chromosome-out scheme may rule out some of the positional influence as observed sites and predicted site may share the same genomic context. However, if the authors' basic assumption is correct in maize, it could be applied to all crop species, like rice and soybean. There are plenty of genomic and population data that the users may be used to validate their assumption. If I understand correctly on the procedure of predicting PNC, between-species validation only using SIFT scores as predictors is doable. I highly encourage the authors to design a validation scheme that may use the model trained in maize to predict PNC in rice. If the predictability maintained at 70% or even lower, it will be very convincing.

*Reply: While leave-one-chromosome-out prediction aimed at avoiding overfitting, all subsequent validations were based on sources of information that were independent from the MSA and the genomic annotations used for training our model. Therefore, validations within maize should provide support for the usefulness of predicted PNC, beyond spurious associations between PNC and genomic annotations in the maize genome.*

*To address the reviewer’s comment and validate predicted PNC in a different genome, we added validation results in sorghum. We chose to validate our model in sorghum, because appropriate data (SIFT score and MAF) was publicly available from a recent publication (Lozano et al. 2021). We trained our model on the whole maize genome, we generate genomic annotations in the sorghum reference genome, and we predicted PNC in sorghum. Then, we compared SIFT score and predicted PNC for average decrease in MAF in sorghum. Results in sorghum are similar to those in maize: more stringent prioritizations by predicted PNC result in a larger decrease in MAF among prioritized SNPs, and prioritizations by predicted PNC result in a larger decrease in MAF, compared to SIFT score. According to these additional validations, our model is capable of prioritizing loci under negative selection, even in a different genome. We added a paragraph in the Results section (ll. 210-229), two figures (Fig. 5 and S6), and a subsection in the Methods section (“Validation in sorghum genome”; ll. 531-549) to describe this analysis and present its results.*

2. Why the authors choose the RF algorithm as predictive model. There are many better models, such as tree-based LightGBM, XGBoost etc. which are more robustness and efficient than RFs. The authors should perform model evaluation and select the optimal one.

*Reply: The focus of the manuscript was on the validation of an approach in which high-resolution genomic annotations (including novel annotations from UniRep) are used to predict PNC. Because proper model comparisons would have required to validate each of the tested models in subsequent analyses, we chose to focus on a commonly used method for fitting nonlinear models from tabular data (RF). To address the reviewer’s comment and assess the benefit from competing methods, we compared RF and gradient-boosted trees (GBT, implemented in the R package xgboost). We show the results of this comparison below (Supplementary table). GBT was more sensitive to hyperparameters (learning rate and number of iterations) compared to RF (number of variables per tree and number of trees), and achieved a slightly higher classification accuracy with an optimal set of hyperparameters (+0.3% increase in classification accuracy for PNC), compared to RF. However, the computational demand for GBT was much larger than for RF (despite very efficient parallelization in the R package xgboost): up to 12 hours to train a GBT model (for each of the 27 sets of hyperparameters tested), compared to < 2 hours to train a RF model in the R package ranger, with parallelization over 40 cores. These results show that GBT may be more accurate than RF, but at much larger computational times.*

*We chose not to include the comparisons between RF and GBT in the manuscript, because we believe that such comparison would require more attention, in a dedicated study.*

*Supplementary table (not included in the manuscript) – Classification accuracy by Random forest and Gradient boosted trees, for different sets of tuning parameters.*

*Classification accuracy for the probability random forest (PRF) or gradient boosted trees (GBT). Methods PRF and GBT were implemented using the R packages ranger and xgboost, respectively. Accuracy: percentage of correct calls, i.e., the percentage of sites in chromosome 8 for which predicted PNC (rounded) equaled observed PNC, over three replicates. Accuracy was weighted to account for imbalance with respect to PNC (see Methods). Hyperparameters: for PRF, number of trees in the forest and number of sampled variables per tree; for GBT, number of trees (iterations) and the learning rate per iteration. In both methods, at least 100 sites were kept at each terminal node in each tree.*

Additionally, the authors should include optimal hyper-parameters determined by grid search-based model tuning.

*Reply: We chose to fit RF models with n_trees_=1000 trees per forest and p_variables_=1/3 of variables sampled per tree, because n_trees_=1000 was our maximal value set for reasonable computational times, and p_variables_=1/3 was the recommended default value for RF (Hastie et al. 2009). To address the reviewer’s comment about tuning, we tuned the PICNC model in leave-one-chromosome-out prediction: “for each left-out chromosome i, optimal hyperparameters n_trees_ and p_variables_ were chosen to maximize weighted classification accuracy, by training in half of the chromosomes (all odd chromosomes when i was even, and vice versa) and validation in the remaining chromosomes (excluding i)”. We agree that tuning should be an integral part of RF model training, and we implemented it for training our PICNC model in leave-one-chromosome-out prediction. However, the default set of parameters (one third of variables sampled per tree and 1000 trees per forest) was consistently chosen as the optimal set of hyperparameters. Therefore, the set of hyperparameters in leave-one-chromosome-out prediction was not modified by tuning. We included a paragraph in the Methods section (ll. 522-530) to describe hyperparameter tuning. We also included sentences in the Results section (ll. 146-150) to describe the sensitivity of RF models to hyperparameters, and we included one figure (Fig. S2) to show the sensitivity of the RF model to hyperparameters, for classification accuracy in chromosome 8.*

3. The SIFT scores ranging from 0.0 (deleterious) to 1.0 (tolerated), which is also developed by the author's lab to predict whether an amino acid substitution affects protein function based on sequence alignments across different species, likely contribute the most when predicting PNC. Is there any correlation between SIFT and PNC? The SNPs at the 99% and 99.9% quantile show higher or lower SIFT score? Whether the SIFT score can be directly used as weights in the MLM model to predict phenotype?

*Reply: We added a plot in Fig. 3 (Fig. 3b) to show the correlation between SIFT score and predicted PNC. As expected, there is a significant negative association between these two proxies for fitness effect (Spearman correlation=-0.70). In our comparison between SIFT scores and predicted PNC (additional paragraph in Results section, ll. 210-229, and Fig. 5), we also showed that more stringent prioritizations of SNPs by predicted PNC point to smaller and smaller subsets of SNPs, among those prioritized by SIFT score (Fig. 5a). In validations by within-species variability (MAF), gene function (gene expression, GO and pathway information) and genomic prediction, we added prioritizations by SIFT scores (Fig. 5, 6, 7, S6, S7, S8; Tables 1 and 2). When prioritizing SNPs above a threshold value (e.g., 90%-percentile) and weighting SNPs in genomic prediction, we used “SIFT conservation” (one minus SIFT score).*

*In genomic prediction validations, we added the results from SIFT score (weighting and prioritization of SNPs by one minus SIFT score). Weighting and prioritizing SNPs by SIFT score did not result in significant gains in prediction accuracy, compared to permuted SNP weights (Table 2, Fig. 7, Fig. S8).*

4. According to Figure 4 and 5, mRNA and proteins of the genes with high PNC (99.9% percentile) tend to express in multiple tissues and high abundance. This result indicates that they are probably housekeeping genes. However, my knowledge of the genes selected by breeding is against this result. The genes targeted by breeding selection are likely non-essential genes, and genetic variations benefiting human-desired trait will not influence the normal development of a plant. The functional enrichment analysis of high-PNC genes is too coarse.

*Reply: To better characterize the genes prioritized by observed/predicted PNC and SIFT score, we conducted a pathway enrichment analysis (using annotations from CornCyc). We added a paragraph in the Results section (ll. 267-278) and a table (Table 1) to show that essential genes tend to be prioritized more. In an additional paragraph in the Discussion section (ll. 363-390), we explained that “on the one hand, evolutionary depth allows us to detect effects of mutations at high spatial resolution; on the other hand, inferences are biased towards certain categories of genes, because fitness effects can only be detected if they are consistent over large evolutionary timescales”. Furthermore, we explained that “to detect effects of single-site mutations on secondary metabolism, other genetic approaches than PICNC may be preferable: analysis of evolutionary divergence or balancing selection, association studies and/or targeted mutagenesis [34,35]”. Therefore, PNC (or SIFT score) may be useful to detect only some of the mutations that impact fitness, and there is no perfect method which can detect all impactful mutations, without any bias (e.g., in QTL mapping: survivorship bias and LD). In the same paragraph in the Discussion section (ll. 363-390), we noted that predicted PNC is nonetheless useful to detect some of the genes and mutations that have high impact on fitness-related traits, as deleterious alleles may be “segregating in natural populations (e.g., variants for carbon-metabolic genes in maize [36]) or fixed by genetic bottlenecks and/or transitions to selfing (e.g., during crop domestication) [32,37]”.*

5. This method could be potentially used to identify causal genes and variants from the GWAS results, as the majority of SNPs significantly associated with an agronomic trait are linked SNPs within a QTL. Is it possible to use the PNC to prioritize the causality of genes to short the candidate list and help identify the causal variants for further functional validation and design of genome editing sites? Multiple previous research in plants and animals have utilized genomic features and functional annotations in a ML system to prioritize genes from GWAS results. The authors may follow these works and it would be a big help if this ideal is successfully implemented.

*Reply: We agree that predicted PNC could help prioritize SNPs with the most evidence for effects on fitness-related traits. In the Discussion section, we mentioned that “PICNC could target few candidate causal variants, for subsequent CRISPR-based base editing (e.g., C-to-T and A-to-G transitions) [39]. Compared to SIFT scores or other proxies for evolutionary constraint, predicted PNC would be especially useful in such applications, by targeting only a handful of sites to be edited simultaneously, for maximal effect on mutational load (Fig. 5) [40,41]” (ll. 385-390). Here, we did not use GWAS hits to validate predicted PNC, because there were few GWAS hits for grain yield in maize (Ramstein et al. 2020). Instead, the reviewer’s suggestion could be followed in a future study, in which validations are performed by mutagenesis. We think that, in such a study, GWAS significance could be combined with predicted PNC, to select the candidate causal variants with the most evidence for fitness effects, as we explain in the last paragraph of the Discussion section (ll. 435-442).*

6. Genomic prediction is the most problematic part. However, this is the most important part, because the authors want to use this result to support their assumption and indicate the usability of the SNPs in breeding. A recent study systematically evaluated 12 ML methods in comparison with rrBLUP for predicting three agronomic traits (flowering time, plant height and grain yield) in six plant species (Azodi et al., 2019). They finally concluded that "No single method performed best across all species and traits, and model tuning for automated optimization of hyper-parameters using grid search was the key to achieving the best performance for all ML methods." If the author wants to include genomic prediction, they have to follow the standard procedure of model evaluation using multiple traits and population.

*Reply: Our genomic prediction validations were conducted in three agronomic traits (days to silking, plant height, and grain yield), and two panels tested in multiple locations in the US (NAM-H and Ames-H). NAM-H consists of 24 families of recombinant inbred lines crossed to the same tester, and Ames-H is a diversity panel of inbred lines crossed to two different testers. In the previous version of the manuscript, all results from genomic prediction validations were shown, but only in supplementary material (Fig. S8). To show all these results in the main text, we included a table (Table 2) to show prediction accuracy from all methods tested, in all panels and traits. We agree that no single method is expected to perform best across populations and traits. Prioritization of nonsynonymous SNPs by predicted PNC is no exception. In the Results section (ll. 328-343), we argue that prioritizing SNPs by predicted PNC is useful if two conditions are met: (i) the trait is related to fitness, such that “some of their causal SNPs are under negative selection”; and (ii) there is enough variability at the prioritized SNPs (“enough statistical information in the training set”). We believe this conclusion is consistent with the statements from Azodi et al. (2019).*

According to Figure 4a, the minor allele frequency (MAF) of SNPs featuring high PNC are mostly below 0.05 or even close to 0. These low-MAF SNPs are usually removed from G2P prediction, as the chance of no polymorphism occurred in training and testing population is too high.

*Reply: Figure 4a shows the average shift in PNC for different MAF values. For example, it shows that predicted PNC is on average higher by 0.10 for SNPs with MAF close to 0. Therefore, Fig. 4a should not be used to evaluate how low the MAF is, among prioritized SNPs. However, it is true that MAF decreases as SNP prioritizations by predicted PNC (or SIFT score) become more stringent (Fig. 5b). In the Results section, we added the average MAF among SNPs prioritized by predicted PNC (> 99.9% percentile) and SIFT score (minimum value of zero): 0.144 and 0.171, respectively. Importantly, it is also worth noting that MAF (based on a reference panel in maize) does not necessarily reflect SNP variability, especially in NAM-H, where the 24 families of recombinant inbred lines had one same recurrent parent in common. In the Results section, we added the mention that “SNPs that are rare in maize are not necessarily rare in NAM-H, because of the half-sib family structure, in which 24 different donor parents are crossed to the same recurrent parent”.*

I also think the slight elevation of accuracy of predicting grain yield is likely attributed to the low MAF SNPs filtered by PNC. Additionally, to ensure the improvement is not a coincidence, the authors need to compile a set of randomly selected SNPs with equal amount of SNPs in different PNC percentile. It has to be done by 100 times of randomization and plot a distribution and include significance test of the comparison.

*Reply: In the first version of the manuscript, significance of genomic prediction accuracy was assessed by comparing the value of genomic prediction accuracy to the values from random permutations. Each random permutation consisted of randomly permuting SNP weights and prioritizing SNPs by these permuted SNP weights, instead of the actual weights. We believe this validation scheme is appropriate to assess whether the genomic prediction accuracy achieved by SNP weights is better than expected by chance. To make the permutation procedure clearer, we added explanations in the Methods section (ll. 636-643): “For each permutation k, ’s were randomly shuffled, and the vector of permuted weights was used to weight and prioritize SNPs, then calculate genomic prediction accuracy as described above. Genomic prediction accuracy from actual weights ’s was deemed significant (P < 0.05) if for all ”. In Figures 7 and S8 (formerly Figures 6 and S7), the distribution of genomic prediction accuracy from random permutations was already shown. We changed the symbols (diamonds for actual weights, circles for permuted weights) to avoid a possible source of confusion. In our validations, we only conducted 20 random permutations, because 100 random permutations would require too much computational time, even after using the qgg R package (Rhode et al. 2020) instead of the regress package, for faster model training.*

Azodi, C.B., Bolger, E., McCarren, A., Roantree, M., de Los Campos, G. and Shiu, S.H. (2019) Benchmarking Parametric and Machine Learning Models for Genomic Prediction of Complex Traits. G3 (Bethesda), 9, 3691-3702.

7. Line 88, "evolutionary constraint (negative selection)". Strictly speaking, evolutionary constraint is not equal to negative selection. Negative selection is to remove deleterious alleles from a population, and evolutionary constraint describe the functional importance of a site. Because the site is too important, it doesn't allow mutations occurred on this site.

*Reply: We removed the mention of negative selection in parentheses, to avoid such confusion.*

*References:*

*Hastie, T., Tibshirani, R., & Friedman, J. (2009). The Elements of Statistical Learning: Data Mining, Inference, and Prediction. Springer, New York, NY.* [*https://doi.org/10.1007/978-0-387-84858-7*](https://doi.org/10.1007/978-0-387-84858-7)

*Lozano, R., Gazave, E., Dos Santos, J. P. R., Stetter, M. G., Valluru, R., Bandillo, N., Fernandes, S. B., Brown, P. J., Shakoor, N., Mockler, T. C., Cooper, E. A., Taylor Perkins, M., Buckler, E. S., Ross-Ibarra, J., & Gore, M. A. (2021). Comparative evolutionary genetics of deleterious load in sorghum and maize. Nature Plants, 7(1), 17–24.* [*https://doi.org/10.1038/s41477-020-00834-5*](https://doi.org/10.1038/s41477-020-00834-5)

*Rohde, P. D., Fourie Sørensen, I., & Sørensen, P. (2020). qgg: an R package for large-scale quantitative genetic analyses. Bioinformatics , 36(8), 2614–2615.* [*https://doi.org/10.1093/bioinformatics/btz955*](https://doi.org/10.1093/bioinformatics/btz955)

**Second round of review**

**Reviewer 1**

The authors have done a good job at addressing my comments. The manuscript has improved a lot and the additional explanations and clarifications make it easier to follow. I also appreciate the inclusions of an additional species to show the wider applicability of the method. I support publication of the revised manuscript.

**Reviewer 2**

This paper proposes a method for identifying causal variants in the maize (plant) genomes - the phylogenetic nucleotide conservation. The metric but has some advantages, and they are able to demonstrate possible association with functionality, making it possible to identify candidate causal variants.

The paper is a revision of a previous submission. The revised version appears to have undertaken extensive revisions based on previous comments by other reviewers, focusing on clarifying and limiting some of their statements. On reviewing the changes, it appears that the reviewers have satisfactorily addressed previous comments.

This paper is a good addition to several others that attempt to develop computational approaches to causal variant characterization/identification. It is particularly useful given its focus on crops, since most of the the other methods are human-centric.

**Reviewer 3**

The authors have addressed all of my previous questions and this version of the the manuscript is much improved. However, the quality of the figure is a bit low, it's better to remake the figures before final publication. Here are some minor suggestions:

Figure 2, the word size in the species tree is too small

Figure 5, word size of the numbers in the venn diagram is too small to read,

Fig 5b, Fig 6, Fig 7, It's better to use the same word size, and rearrange the panels. They are too small to read.

**Authors Response**

**Point-by-point responses to the reviewers’ comments:**

Reviewer #3:

The authors have addressed all of my previous questions and this version of the the manuscript is much improved. However, the quality of the figure is a bit low, it's better to remake the figures before final publication. Here are some minor suggestions:

Figure 2, the word size in the species tree is too small

Figure 5, word size of the numbers in the venn diagram is too small to read,

Fig 5b, Fig 6, Fig 7, It's better to use the same word size, and rearrange the panels. They are too small to read.

*Reply: All comments from the editor and reviewer 3 were addressed.*

*Word size in the species tree was increased in Figure 2.*

*Word size was increased in Fig. 5A.*

*Figure 5, 6, 7 and S7 were arranged vertically instead of horizontally to avoid restrictions on their size.*
